# Digital Gene Expression Analysis Based on *De Novo* Transcriptome Assembly Reveals New Genes Associated with Floral Organ Differentiation of the Orchid Plant *Cymbidium ensifolium*

**DOI:** 10.1371/journal.pone.0142434

**Published:** 2015-11-18

**Authors:** Fengxi Yang, Genfa Zhu

**Affiliations:** Guangdong Key Laboratory of Ornamental Plant Germplasm Innovation and Utilization, Environmental Horticulture Research Institute, Guangdong Academy of Agricultural Sciences, Guangzhou, 510640, P. R. China; University of Naples Federico II, ITALY

## Abstract

*Cymbidium ensifolium* belongs to the genus *Cymbidium* of the orchid family. Owing to its spectacular flower morphology, *C*. *ensifolium* has considerable ecological and cultural value. However, limited genetic data is available for this non-model plant, and the molecular mechanism underlying floral organ identity is still poorly understood. In this study, we characterize the floral transcriptome of *C*. *ensifolium* and present, for the first time, extensive sequence and transcript abundance data of individual floral organs. After sequencing, over 10 Gb clean sequence data were generated and assembled into 111,892 unigenes with an average length of 932.03 base pairs, including 1,227 clusters and 110,665 singletons. Assembled sequences were annotated with gene descriptions, gene ontology, clusters of orthologous group terms, the Kyoto Encyclopedia of Genes and Genomes, and the plant transcription factor database. From these annotations, 131 flowering-associated unigenes, 61 *CONSTANS-LIKE* (*COL*) unigenes and 90 floral homeotic genes were identified. In addition, four digital gene expression libraries were constructed for the sepal, petal, labellum and gynostemium, and 1,058 genes corresponding to individual floral organ development were identified. Among them, eight MADS-box genes were further investigated by full-length cDNA sequence analysis and expression validation, which revealed two *APETALA1/AGL9*-like MADS-box genes preferentially expressed in the sepal and petal, two *AGAMOUS*-like genes particularly restricted to the gynostemium, and four *DEF*-like genes distinctively expressed in different floral organs. The spatial expression of these genes varied distinctly in different floral mutant corresponding to different floral morphogenesis, which validated the specialized roles of them in floral patterning and further supported the effectiveness of our *in silico* analysis. This dataset generated in our study provides new insights into the molecular mechanisms underlying floral patterning of *Cymbidium* and supports a valuable resource for molecular breeding of the orchid plant.

## Introduction

*Cymbidium* is a very economically important flowering genus of the orchid family (Orchidaceae) in China, Japan, Korea, and Southeast Asia [1, 2]. *Cymbidium ensifolium*, which belongs to the subgenus *Jensoa* of genus *Cymbidium*, is a prevalently potted plant and cut flower cultivated in China [3, 4]. Its unique floral organ includes three sepals in the first whorl, three petals in the second whorl and productive parts in the center of the flower. The sepals and petals together are called the tepals. Among them, two of the petals are similar to each other and resemble unmodified sepals, while the third is highly modified and is called the labellum (or lip). Its shape is ovate to triangular and often has a kinked to undulated margin. The male and female productive organs are highly fused to form a gynostemium (or column), which evolved through complete fusion of the style, stigma and staminal filament, and has four pollinia on a semi-circular viscidium [5, 6]. Despite this spectacular flower morphology, various mutations occur frequently in the orchid family and greatly diversify the floral morphology [7–12]. For example, the gynostemium is replaced by a new emerged flower in the multi-tepals mutant, which continues to produce sepals, petals, and gynostemium centripetally, whereas in the non-gynostemium mutant, the gynostemium is absent and replaced by new sepals in the center part of the flower. Owing to their highly specialized and diversified floral morphology, these mutants are much more valuable and attract more horticultural and commercial attention. However, the functional genomic studies and the gene discovery associated with floral patterning regulation remains greatly limited in the *Cymbidium* orchid due to the polyploidy genomes and long juvenile phases [13, 14]. Despite their apparent importance, molecular and genetic approaches to orchid flower development and evolution are still in their infancy.

In recent years, next-generation sequencing (NGS) technologies have provided powerful tools for high-throughput sequence determination. Various NGS-based RNA-sequencing modes have made obtaining massive sequences with enormous depth and coverage possible, thus enabling the discovery of novel genes [15–17]. An increasing number of studies have highlighted the utility of cDNA sequencing for discovering candidate genes associated with floral development, floral scent production, or flowering time in various orchid species (such as genera *Phalaenopsis*, *Oncidium*, *Ericina*, *Dendrobium*) in the absence of a genomic sequence [18–21]. For example, using genetic recourses, four *APETALA3-like* genes, *PeMADS2*, *PeMADS3*, *PeMADS4*, and *PeMADS5*, were found to display distinct floral morphogenetic roles in various floral organs of *Phalaenopsis*. Additionally, *PeMADS6* was found to be involved in petaloid formation and correlated with ovary development [22, 23]. In *Oncidium Gower Ramsey*, *OMADS1* was isolated and characterized as a functional gene promoting flower transition and formation by acting as an activator of *FT* and *SOC1*, and was able to strongly interact with *OMADS3*, which influenced flower formation and floral initiation [24]. In *Dendrobium crumenatum*, B function genes *DcOAP3A/B* and *DcOPI* could form heterodimers and further interact with *DcOSEP* to form higher protein complexes and mediate flower formation and development [25]. However, similar studies in the genera *Cymbidium* are limited, and the number of genes in current databases is still not enough to support further functional genomics studies when compared with other plant species [26, 27]. A very recent result from next-generation sequencing generated 51,696 genes for *C*. *ensifolium* [26]. Nevertheless, little functional genomics research has been performed, and the molecular aspects of flowering control and floral organ development of *C*. *ensifolium* remain largely unknown.

To obtain an overview of the gene expression information and provide plenty of functionally characterized candidate genes directly associated with floral development in *Cymbidium*, we sequenced the floral transcriptome of *C*. *ensifolium ‘*tianesu’, which is a typical *C*. *ensifolium* cultivar, well-known for its valuable horticultural traits, including large flowers and varied floral morphology [1, 3]. A total of 10.9 Gb clean sequence data and 111,892 unigenes were generated, and an informative EST dataset was obtained, which can be used as an important resource for molecular breeding, marketable traits, and investigating various biological process in *Cymbidium*. To facilitate identifying sets of genes involved in individual floral organ development, we developed DGE tags derived from different floral organs. More than 1,000 genes with highly differential expression levels were identified as candidates connected with floral pattern regulation. Among them, we concentrated on the MIKC-type transcription factors and found 16 MADS-box genes expressed preferentially among the sepal, petal, labellum and gynostemium. Phylogenetic analysis demonstrated that 14 of these MADS-box genes clustered well with the floral homeotic genes isolated from other orchid species. Eight of them were further characterized by gene cloning and expression analysis. The result from quantitative RT-PCR suggested distinct roles of them on floral organ identity, which indicated that we developed a useful transcriptome database for identifying the candidate genes responding to the individual floral organ development. This study provides new insights into the molecular mechanisms underlying floral patterning and broadens our understanding of flower development in *Cymbidium*.

## Materials and Methods

### Plant materials and growth conditions

The plants of *C*. *ensifolium* ‘tianesu’ used in this study were artificially cultivated and collected from the cultivation base of Environmental Horticulture Research Institute, Guangdong Academy of Agricultural Sciences, China. All of the plants were grown and maintained in pots in a greenhouse at day/night temperatures of 26/23°C under a 16-h light /8-h dark photoperiod.

### cDNA preparation and Illumina sequencing

For transcriptome sequencing, the cDNA library was prepared from an equal mixture of RNAs isolated from flower buds and mature flowers of *C*. *ensifolium* ‘tianesu’. For the Digital Gene Expression (DGE) analysis, we constructed four independent cDNA libraries for the sepal, petal, labellum, and gynostemium separately. Total RNA was extracted from approximately 0.5 g of each tissue using the TRIzol reagent (Invitrogen). Individual mRNAs were purified from total RNA using the Oligotex mRNA Midi Kit (QIAGEN) and quantified using a NanoDrop 2000 spectrophotometer (Thermo Scientific) to generate the cDNA library according to the Illumina manufacturer’s instructions. Briefly, fragmentation buffer was added to interrupt mRNA to short fragments. Random hexamer primers were added to these short fragments to synthesize the first-strand cDNA. The second-strand cDNA was synthesized using the SuperScript double-stranded cDNA synthesis kit (Invitrogen) and purified with a QiaQuick PCR extraction kit (QIAGEN).The double-stranded cDNA was sequenced using illumina Hiseq 2500 platform.

### Sequence assembly and annotation

The resulting raw sequence reads with weak signal or low quality were screened and trimmed by GS FLX pyrosequencing software, to yield a final dataset comprised of high-quality (HQ) (>99.5% accuracy of single-base reads) sequences. Prior to assembly, primer and adapter sequences were trimmed from the HQ dataset, and sequences shorter than 50 bp were removed. The remaining data were assembled into unique sequences (including contigs) using trinityrn (http://trinityrnaseq.sourceforge.net/analysis/extract_proteins_from_trinity_transcripts.html.).

For unigene annotations, the genes in which could find protein coding sequences were searched against the NCBI non-redundant protein (Nr) database (http://www.ncbi.nlm.nih.gov) and the SwissProt protein database (http://www.expasy.ch/sprot) using the BLASTP algorithm with an E-value cut-off of 10^−5^. The remaining sequences were searched against public databases, using the BLASTX algorithm with an E-value cut-off of 10^−5^ [28].

Gene ontology (GO) classification was obtained by GO terms in the database (http://www.geneontology.org/). The unigene sequences were also aligned to the COG database to predict and classify functions [29]. Pathway assignments were made according to the Kyoto Encyclopedia of Genes and Genome (KEGG) mapping project, (http://www.genome.jp/) [30]. Enzyme commission (EC) numbers were assigned to unique sequences that had BLASTX scores with an E-value cut-off of 10^−5^ as determined by searching the KEGG database. The unique sequences were allocated to specific biochemical pathways according to the corresponding EC distribution in the KEGG database.

For identification of transcription factor-related unigenes in our *C*. *ensifolium* dataset, the protein sequences of predicted transcription factors for rice were downloaded from the Plant Transcription Factor Database (PTFDB; http://planttfdb.cbi.pku.edu.cn/). These protein sequence set of each transcription factor family from rice was BLAST against the clusters and singletons in our dataset with use of the TBLASTN programs. Sequence similarity was considered significant at E-value < 10^−5^.

### Expression analysis of DGE tags

The gene expression was calculated by the numbers of reads mapped to the reference sequence. To map the DGE tags, the sequenced raw data were filtered to remove low quality tags (including tags with an unknown nucleotide “N”, empty tags, and tags with only one copy number). Clean tags were mapped to our transcriptome reference database using TopHat [31]. No more than two read-mismatches and read-gap-length were allowed in the alignment.

The gene expression level was calculated by the value of FPKM(Fragments Per Kilobase of transcript per Million mapped reads. RPKM = total exon Fragments/ mapped Fragments (millions) * exon length (KB). To compare the differences in gene expression among different floral organs, the tag frequency in each DGE libraries was statistically analyzed according to the method described by Audic and Claverie. We used FDR < 0.001 and an absolute value of |log2(Fold change)|>1, q_value<0.05 as the threshold to determine the significant difference in gene expression [32].

### Gene ontology and pathway enrichment analysis of DEG

The differentially expressed genes were annotated using GO and KEGG enrichment analyses according to a method similar to that described by Wang [32], which firstly mapped all DEGs to GO terms (or KEGG pathways) in the database (http://www.geneontology.org/, http://www.genome.ad.jp/), calculating gene numbers for every term (or pathway). Followed by a hypergeometric test to find the significantly enriched terms in DEGs compared with the genome background. We take the corrected P-value ≤ 0.05 or Q-value ≤ 0.05 as a threshold for the significantly enriched GO terms or KEGG pathways, respectively, in DEGs.

### Real-time quantitative RT-PCR (qRT-PCR) analysis

When determining the circadian rhythm of the *COL* genes, leaves from one-year-old *C*. *ensifolium* plants were collected in 4-h intervals for 24 h after the start of light exposure in LD conditions (16-h light /8-h dark). Each sample was collected from three plants. For the expression of MADS-box genes, mature flowers were dissected into their individual floral organs (sepals, petals, labellums and gynostemiums). Total RNAs were extracted using TRIzol RNA extraction method (Tiangen, China) and reverse transcribed into cDNA using PrimeScritH RT reagent kit with gDNA Eraser (Perfect Real Time) (Takara, China). The qRT-PCR was performed with a Bio-Rad CFX-96 RealTime PCR System (Bio-Rad, US) in a final volume of 20 μl containing 2 μl cDNA, 10 μl SYBR premix Ex taqTM (Takara, Japan), 0.4 μl 10 mM the forward and reverse primers each, and 7.2 μl RNase-free water. The thermal cycling conditions were as follows: 95°C 5 min, 40 cycles at 95°C for 5 s for denaturation and 59.8 C for 25s for annealing and extension. Ubiqutin was used as an internal control for normalization to make a comparison of gene expression level between the accessions. The primers designed with the software Primer 5.0 were listed in S1 Table.

## Results and Discussion

### Transcriptome sequencing and assembly

**Fig 1:**
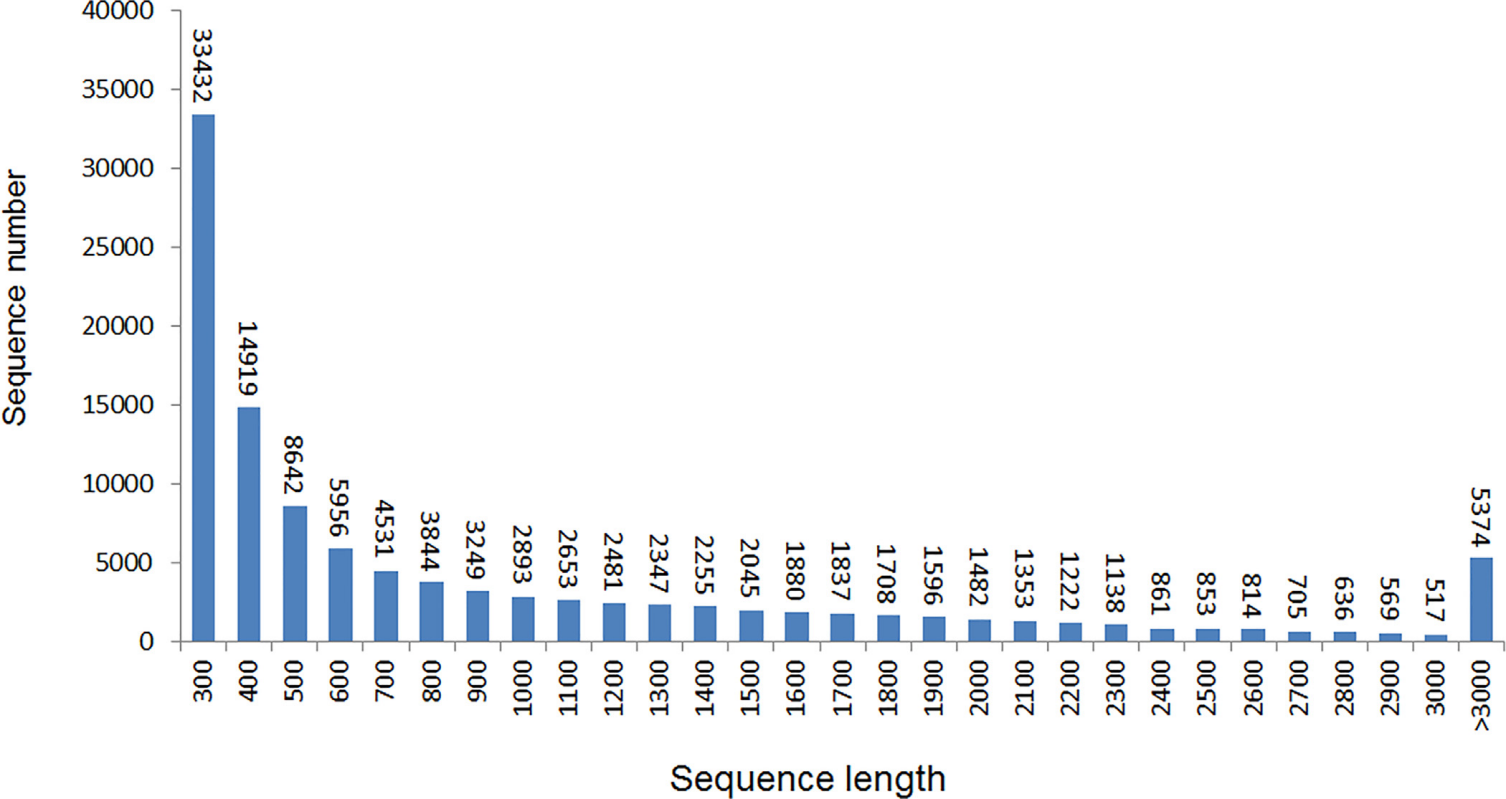
The length distribution of assembled unigenes. The x-axis represents the sequence length in base pairs. The y-axis represents the unigenes number.

We pooled the cDNA library from an equal mixture of RNAs isolated from flower buds and mature flowers. After sequencing, 109,366,378 reads (89.92% of the raw data) that passed quality control were entered into the Trinity assembly process and yielded 136,553 contigs, with 148,968,762 total residues. These contigs were further assembled into 111,892 unigenes, including 1,227 clusters and 110,665 singletons, with a minimum unigene size of 201 bp, a maximum size of 16,814 bp, an N50 of 1,697 bp and a mean length of 932.03 bp (Table 1). As shown in Fig 1, the assembly produced a substantial number of large unigenes: 14,154 unigenes were > 2,000 bp in length and 34,450 unigenes were > 1,000 bp, although most unigenes were between 300 and 500 bp in length. To validate the effectiveness of our de novo assembly, the unigenes generated in this study were BLAST against the previously available ESTs derived from *C*. *ensifolium*. Results showed that among 111,892 unigenes, 101,486 (90.7%) matched the previously published ESTs. And the remaining 10,406 unigenes that did not matched probably represent the sequences newly identified in *C*. *ensifolium*. These massive sequences within our dataset provide more comprehensive gene expression information that could be used as a valuable resource for exploring the genetic diversity of *C*.*ensifolium* and for comparative genomic studies among orchid plants.

**Table 1:** Summary of sequencing and *de novo* assembling of the floral transcriptome in *C*. *ensifolium*.

Summary of *C*. *ensifolium* Floral Transcriptome	
Raw reads	121,626,996
Clean reads	109,366,378
Total clean nucleotides	10,924,593,174 bp
Total contigs	136,553
Total residues	148,968,762 bp
Total unigenes	111,892
Smallest unigene	201 bp
Largest unigene	16814 bp
Average length	932.03 bp

### Functional annotation and classification of the transcriptome

**Fig 2:**
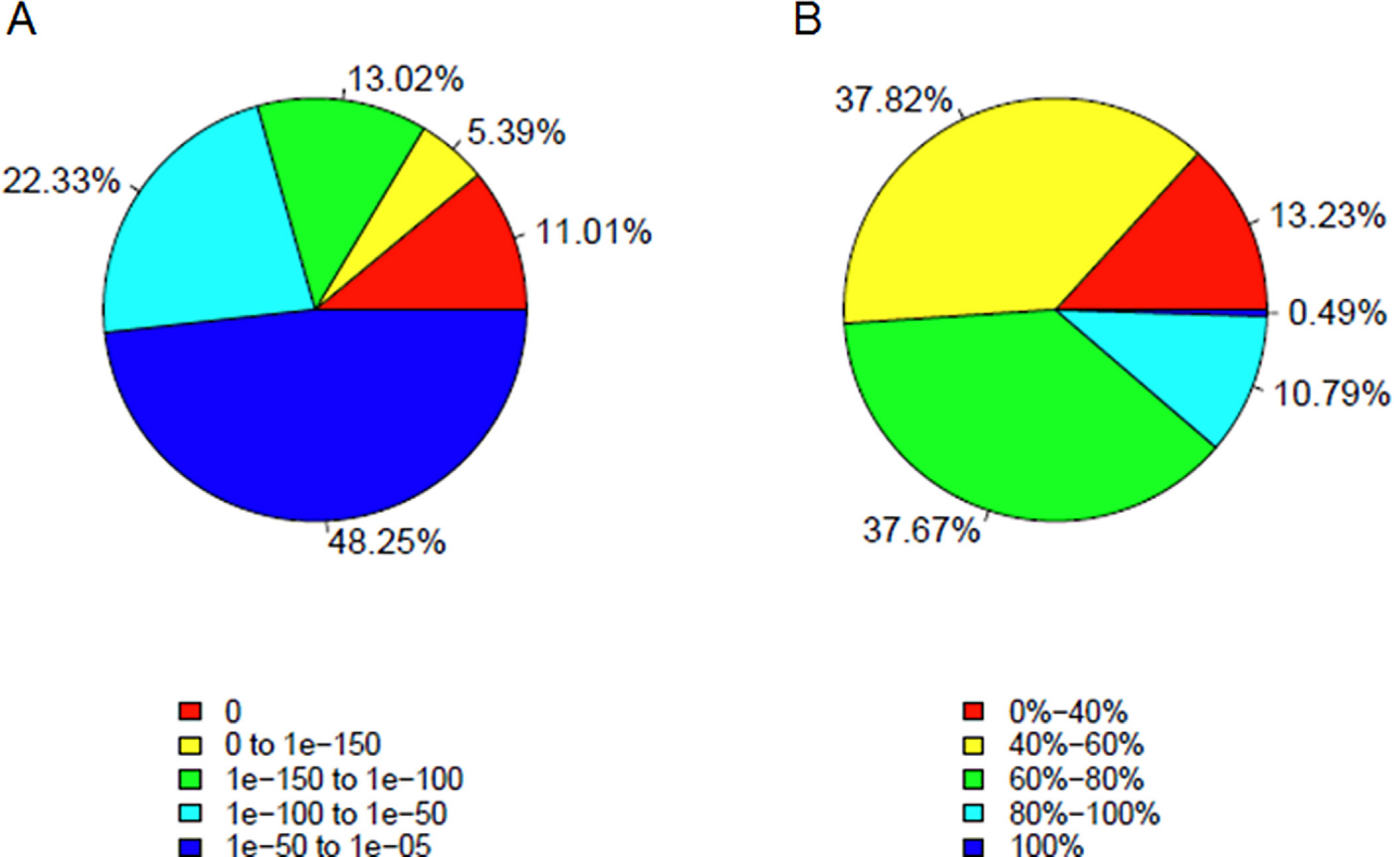
Characteristics of homology search of the unigenes in Nr database.

To functionally annotate the *C*. *ensifolium* floral transcriptome, the unigene sequences were used as query in a BLAST search against the NCBI non-redundant (Nr) protein, gene ontology (GO), clusters of orthologous group (COG), and the Kyoto Encyclopedia of Genes and Genomes (KEGG) databases using a cut off E-value of e-5. Consequently, a total of 62,139 unigenes (55.53% of all the assembled unigenes) provided a significant BLAST result (Table 2) and most matches were found in the NCBI-Nr database (47,285 or 42.26%). According to the E-value distribution of the top hits in the databases, 51.75% of the matched sequences showed strong homology (< 1.0e^-50^), while 48.25% of the matched sequences showed moderate homology (between 1.0e^-5^ and 1.0e^-50^) (Fig 2A). The identity distribution pattern showed that 11.28% of these alignments had a similarity higher than 80%, 37.67% between 60% and 80%, and 51.05% lower than 60% (Fig 2B).

**Table 2:** Summary of the blast hits against the known protein database.

	Num	Percent
Total	62,139	55.53%
Nr	47,285	42.26%
ORF	30,091	26.89%
COG	43,018	38.45%
GO	32,632	29.16%
KEGG	21,461	19.18%

**Fig 3:**
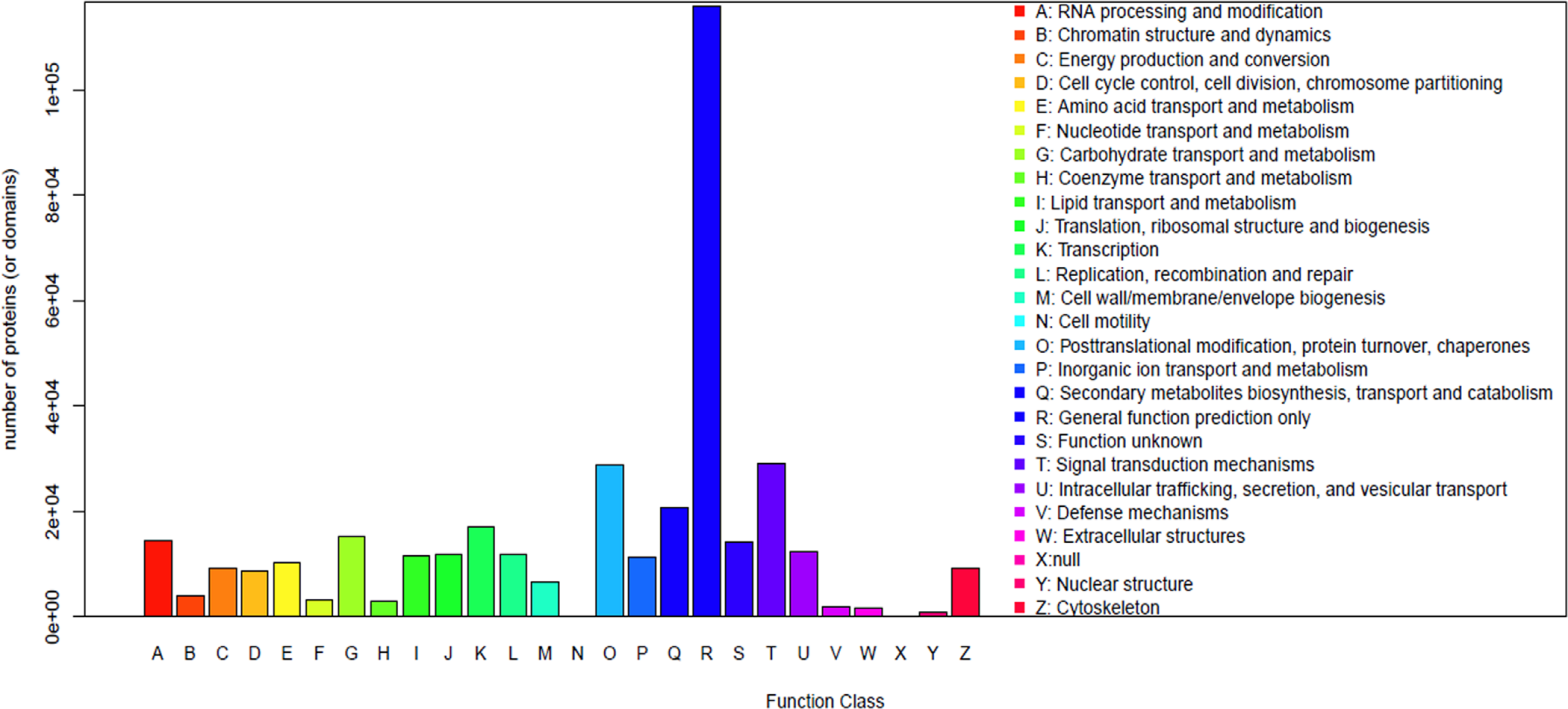
COG function classification of assembled unigenes.

In the COG classification, 43,018 unigenes (38.45%) were classified into 25 functional classifications (Fig 3). ‘General function prediction’ was dominant (11,569), followed by ‘Posttranslational modification’ (6,795), ‘Signal transduction’ (5,266) and ‘Secondary metabolites biosynthesis’ categories (2,199). ‘RNA processing and modification’, ‘Carbohydrate transport and metabolism’, and ‘Transcription’ also shared a relatively high-percentage of genes among the categories. Only a few genes matched the terms ‘Nuclear structure’ and ‘Extracellular structures’.

**Fig 4:**
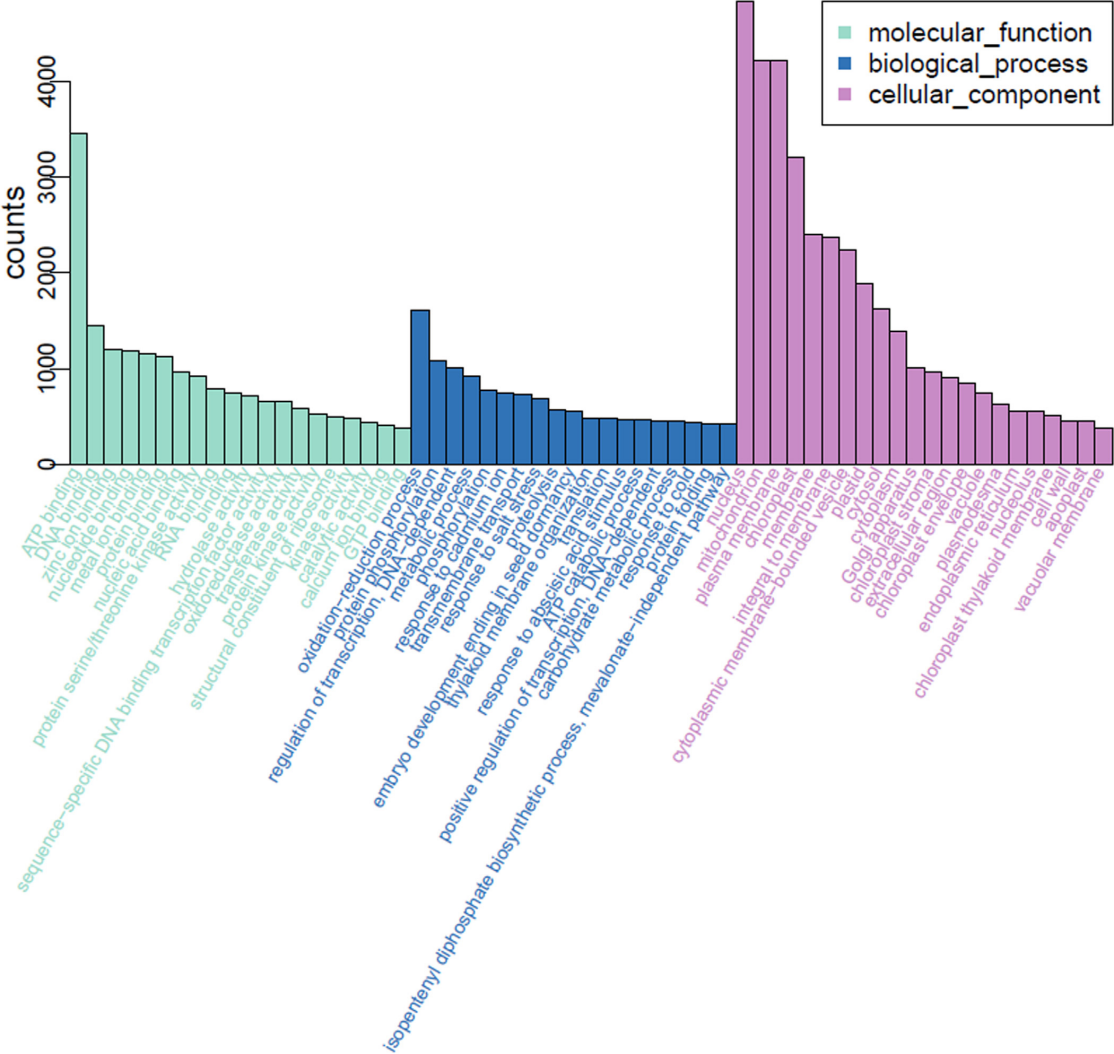
GO classification of unigenes of assembled unigenes.

When aligned to the GO system, 32,632 (29.16%) unigenes were categorized into 61 functional groups (Fig 4). Of which, 20 GO terms were related to ‘molecular functions’, 19 were related to ‘biological process’, and 22 were related to ‘cellular component’. The three largest percentages of genes within these three function categories were ‘ATP binding’ (9,585, 48.3%), ‘oxidation-reduction process’ (14,119, 71.2%) and ‘nucleus’ (10,809, 54.5%). In addition, we noticed that a high-percentage of genes came from the ‘mitochondrion’ and ‘plasma membrane’ groups in the ‘cellular component’ category and a relative high percentage of genes came from the ‘binding’ and ‘protein phosphorylation’ groups in the ‘molecular functions’ and ‘biological process’ categories, respectively. However, only few genes came from the terms ‘extracellular region part’ and no gene came from the terms ‘metallochaperone’, ‘nutrient reservoir’, or ‘translation regulator’.

We also mapped the assembled unigenes to the reference canonical pathways in KEGG, including metabolism, genetic information processing, environmental information processing, cellular processes, and organism systems (http://www.genome.jp/kegg/pathway.html). In total, 21,461 unigenes (37,583,629 reads) were mapped into 329 KEGG pathways representing 19.18% of the assembled unigenes. Among these, 3,776 unigenes were related to metabolism, which represented the most mapped sequences. Additionally, 2,688 unigenes corresponded to genetic information processing, 1,238 unigenes belonged to organism systems, 1,094 unigenes were classified as cellular processes, and 823 unigenes mapped to environmental information processing (Table 3). The presence of genes for all of these essential biological processes suggests that these sequences account for most of the comprehensive transcriptome. These pathways provide valuable resources for investigating specific biological processes during *C*. *ensifolium* floral development. Among these, the circadian clock is an important part of the photoperiod pathway controlling flower initiation. A circadian rhythm was found in the KEGG pathway and its detailed metabolic pathway is shown in S1 Fig. The 66 unigenes involved (S2 Table) will facilitate further studies on the effects of the photoperiod pathway on flowering control in *C*. *ensifolium*.

**Table 3:** Summary of the KEGG pathway and their corresponding gene number.

KEGG Pathways	Sub-pathways of KEGG Pathway	Number of unigenes	Number of reads
**Cellular Processes**		1,094	1,693,012
	Transport and catabolism	595	1,187,266
	Cell motility	113	123,121
	Cell growth and death	414	417,722
	Cell communication	161	268,922
**Genetic Information Processing**		2,688	4,615,076
	Transcription	436	618,315
	Translation	1,103	2,358,454
	Folding, sorting and degradation	845	1,804,603
	Replication and repair	484	203,113
**Environmental Information Processing**		823	986,367
	Membrane transport	149	96,975
	Signal transduction	663	874,444
	Signaling molecules and interaction	13	14,951
**Organismal Systems**		1,238	1,772,131
	Immune system	334	598,690
	Endocrine system	269	512,321
	Circulatory system	99	103,034
	Digestive system	245	154,968
	Excretory system	140	177,267
	Nervous system	283	429,307
	Sensory system	46	33,599
	Development	46	50,614
	Environmental adaptation	309	461,208
**Metabolism**		3,776	7,328,381
	Carbohydrate metabolism	907	2,176,302
	Energy metabolism	840	2,277,509
	Lipid metabolism	574	1,700,740
	Nucleotide metabolism	459	354,067
	Amino acid metabolism	644	1,498,932
	Metabolism of other amino acids	250	682,150
	Glycan biosynthesis and metabolism	310	152,222
	Metabolism of cofactors and vitamins	512	473,178
	Metabolism of terpenoids and polyketides	272	419,236
	Biosynthesis of other secondary metabolites	22	566,666
	Xenobiotics biodegradation and metabolism	104	398,766
	Total	21,461	37,583,629

### Identification of transcription factors

**Fig 5:**
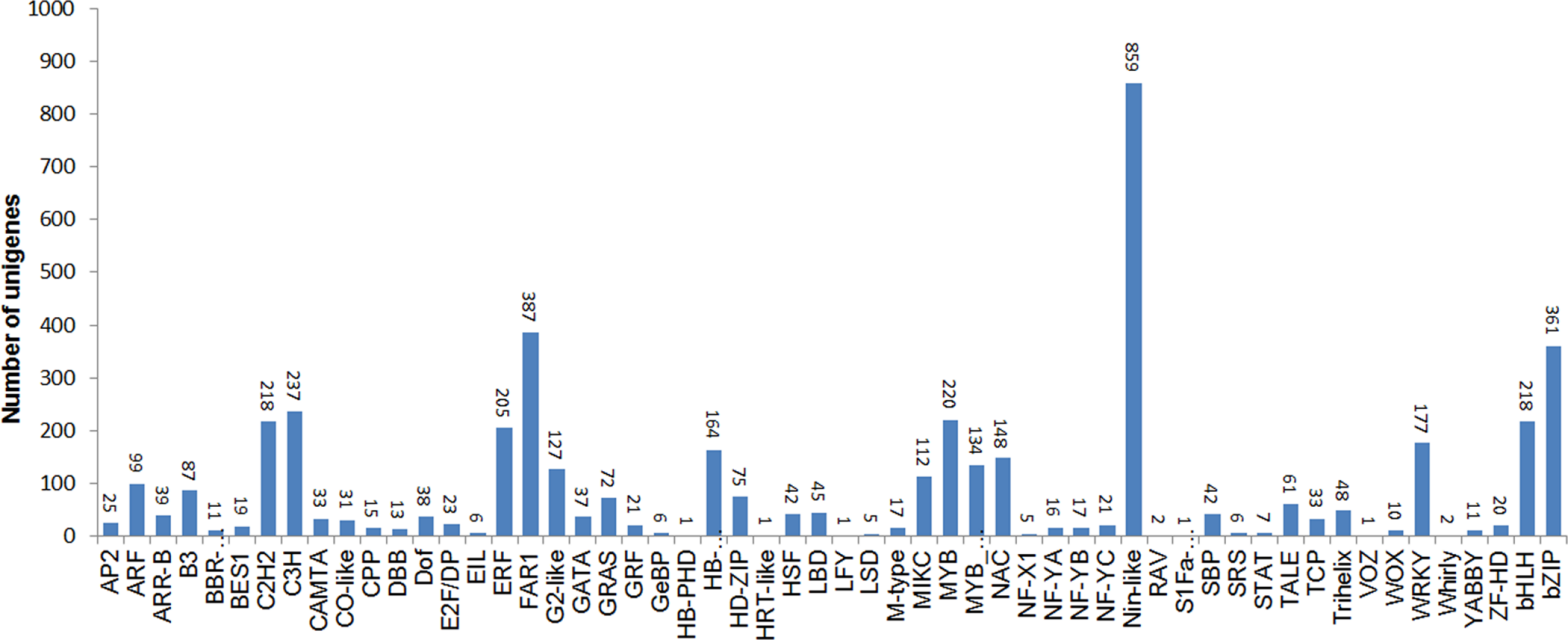
Predicted transcription factors of *Cymbidium ensifolium*.

Transcriptional regulation of floral development has been well documented in *Arabidopsis* and many other non-model plants, but little has been known in *C*. *ensifolium*. In this research, we identified the transcription factors from the transcriptome of *C*. *ensifolium* by aligning rice transcription factor sequences (downloaded from http://planttfdb.cbi.pku.edu.cn/) against the EST dataset. In a total, 4,632 unigenes were identified, belonging to 56 putative transcription factor families using Blastx with a cut off E-value of below 10^−5^ (Fig 5).

Among the all putative transcription factor encoding genes, the Nin-like family, which is identified as core symbiotic genes required for establishing symbiosis between legumes and nitrogen fixing bacteria, collectively called the Rhizobium, are the most abundantly expressed transcription factor genes, accounting for 17.5% of the overall transcription factors. In contrast, expression studies in *Arabidopsis* and rice genomes found barely nine and three Nin-like proteins, respectively, suggesting that Nin homologs constitute small gene families in these model plants [33]. The huge number of Nin-like homologs found in *Cymbidium*, which is greatly different from in *Arabidopsis* and rice, might be related to the importance of their functions in symbiosis and nitrogen metabolism in orchidaceae [33, 34]. In addition, 387 and 361 genes were identified as FAR1 and bZIP respectively. C3H (237), MYB (220), C2H2 (218), bHLH (218), ERF (205), G2-like (167), WRKY (177), NAC (148), and MYB-related (134) families were also found. These nine families formed ~30% of the expressed transcription factors, and the number in each family was more than in *Arabidopsis* and the rice genomes. Whole-genome duplication occurred in early monocots resulting in polyploidy, gene duplication, gene loss and rearrangements. This may also be the reason why greater numbers of transcription factors are found in the orchid genome, as suggested by the presence of the four *AP3*-like paralogs that form the basis for the complicated floral morphologies of *Phalaenopsis* [18]. Paralogous genes are present in at least four out of the five subfamilies of the orchidaceae, suggesting that some genes may have been undergone gene duplication. However, no more than five members of the transcription factor families Whirly, LFY, RAV, VOZ, HRT-like, and HB-PHD were detected. This result is consistent with the previous report that few members of these six families were found in *Phalaenopsis* [35]. This might result from the rare expression of these genes. Large-scale deeper transcriptome sampling and sequencing efforts will help identify genes related to transcription factor families in orchids.

### Functional genes involved in floral development and flowering

Since one of our aims was to identify genes that are responsible for floral traits and flower development, we specifically searched for the orthologs of genes and gene families that are known to be involved in the ABCDE flower developmental model of *Arabidopsis*. According to the unigene annotations, we obtained 90 involved genes: 43 unigenes were identified as class A genes (eg. *AP1*) homologs, 14 unigenes as class B genes (*AP3* and *PI*) homologs, 22 unigenes as class C genes (*AG*) homologs, and 6 and 5 genes as putative orthologs of class D (*SHP* and *STK*) and E genes (*SEP* and *AGL*), respectively (S3 Table). In addition to these, we found a total of 129 putative MADS-box transcription factors expressed in the floral transcriptome, including the genes without floral homeotic functions that are members of the MIKC, Mα, Mβ, Mγ, and Mδ subfamilies (S4 Table). Considering that the MADS-box genes are responsible for flower development, this comparative identification of the MADS-box unigenes could be used as an important resource for floral development and flower organ formation studies in the future.

Additionally, the gene orthologs governing time to flower were also analyzed. A total of 131 unigenes that showed homology to the known flowering-related genes in other plants were identified, and some are listed in Table 4. These include floral integrator pathway genes related to *FLOWERING LOCUS T* (*FT*), *APETALA2* (*AP2*), and *SUPPRESSOR of OVEREXPRESSION of CONSTANS1* (*SOC1*), floral meristem identity genes *LEAFY*(*LFY*) and *APETALA1*(*AP1*), and vernalization pathway genes related to *VERNALIZATION* (*VRN1*), *FLOWERING LOCUS C* (*FLC*) and *FLOWERING LOCUS D (FLD*), as well as a number of genes responding to the photoperiod pathway, such as *PHYTOCHROME A* (*PHYA*), *PHYTOCHROME B* (*PHYB*), *PHYTOCHROME INTERACTING FACTOR 3* (*PIF3*), *EARLY FLOWERING 3* (*EIF3*), *LATE ELONGATED HYPOCOTYL* (*LHY*), *CIRCADIAN CLOCKASSOCIATED 1* (*CCA1*), *CONSTITUTIVE PHOTOMORPHOGENIC 1*, (*COP1*), *TIMING OF CAB EXPRESSION 1* (*TOC1*), *GIGANTEA* (*GI*), *CHALCONE SYNTHASE* (*CHS*), and *CONSTANT* (*CO*).

**Table 4:** Representatives of putative flowering-time genes in *C*.*ensifolium*.

	Gene ID	Homologous gene	Gene Nr-ID	Identidity Nr_top (%)
**Photoperiod pathway**	Comp58987	PHYA	XP_002278610.1	68.4
	Comp54211	PHYB	XP_003558068.1	71.7
	Comp5851		XP_004295077.1	70.8
	Comp35281	PIF3	XP_002276198.2	38.6
	Comp53073		XP_002276198.2	37.4
	Comp52955	EIF3	ABL11477.1	38
	Comp50981	LHY	NP_001131529.2	34
	Comp52658		BAC99516.1	76.5
	Comp57367		BAC99516.1	37.8
	Comp57910	CCA1	XP_004306608.1	80.6
	Comp35965	COP1	BAA94422.1	60.2
	Comp54784	TOC1	ADO51646.1	62.5
	Comp48883	GI	AEB35426.1	72.4
	Comp56138		ADP92454.1	80.3
	Comp27950	CHS	AGJ50587.1	83
	Comp48799		NP_001064831	59.3
	Comp37213	CO	NP_001047975.1	87.2
	Comp51149		NP_001057441.1	86.7
**Floral integrator pathway**	Comp26556	FT	AFS17371.1	54.5
	Comp39786		ADP89905.1	78.3
	Comp41343		ADW76861.1	98.5
	Comp44994		AFU08240.1	73.8
	Comp48314		ADP89470.1	63.8
	Comp301941		BAD01612.1	86.3
	Comp36081	AP2	AGI62045.1	72.8
	CL2711Contig1		ABU68665.1	48.9
	CL2228Contig1		AGI62044.1	72
	Comp43524		AGI62041.1	82.1
	Comp51324		AGI62047.1	80.2
	Comp26637		AGK07583.1	68.6
	Comp47321		AGK07583.1	84.8
	Comp55126		NP_001236377.1	84
**Floral meristem identity**	Comp45071	SOC1	AFQ31623.1	66.4
	Comp47662		AFQ31623.1	74.8
	Comp58027	AP1	AFQ31623.1	99.6
	Comp51302	LFY	AGE45851.1	97.8
**Vernalization pathway**	Comp45077	VRN1	AEV22381.1	58.9
	Comp59230	FLC	ACZ26524.1	26.1
	CL346Contig1	FLD	AAX51267	58.7
	Comp58957		NP_001148070.1	50

### Identification of CONSTANS-like gene family

CONSTANS (CO) is a transcription factor that promotes flowering by inducing the expression of the direct downstream genes FT and SOC1 [36, 37]. In many plant species, a large family of *CONSTANS-LIKE* (*COL*) genes has been identified and plays a central role in the photoperiod response pathway by mediating between the circadian clock and the floral integrators [36–38]. To identify the *COL* genes in *C*. *ensifolium*, the *Arabidopsis* CO and COL amino acid sequences were used to screen for homologs in our dataset using tBLASTN. In total, 61 putative COL homologs were identified and are listed in S5 Table. After translation, sets of these proteins grouped together and showed homology with the COL proteins from *Arabidopsis*. As shown in S2 Fig, 16 COL homologs of *C*. *ensifolium* were clustered well with 17 *Arabidopsis* COL proteins and can be divided into three subgroups as previously defined in *Arabidopsis* [36, 37].

**Fig 6:**
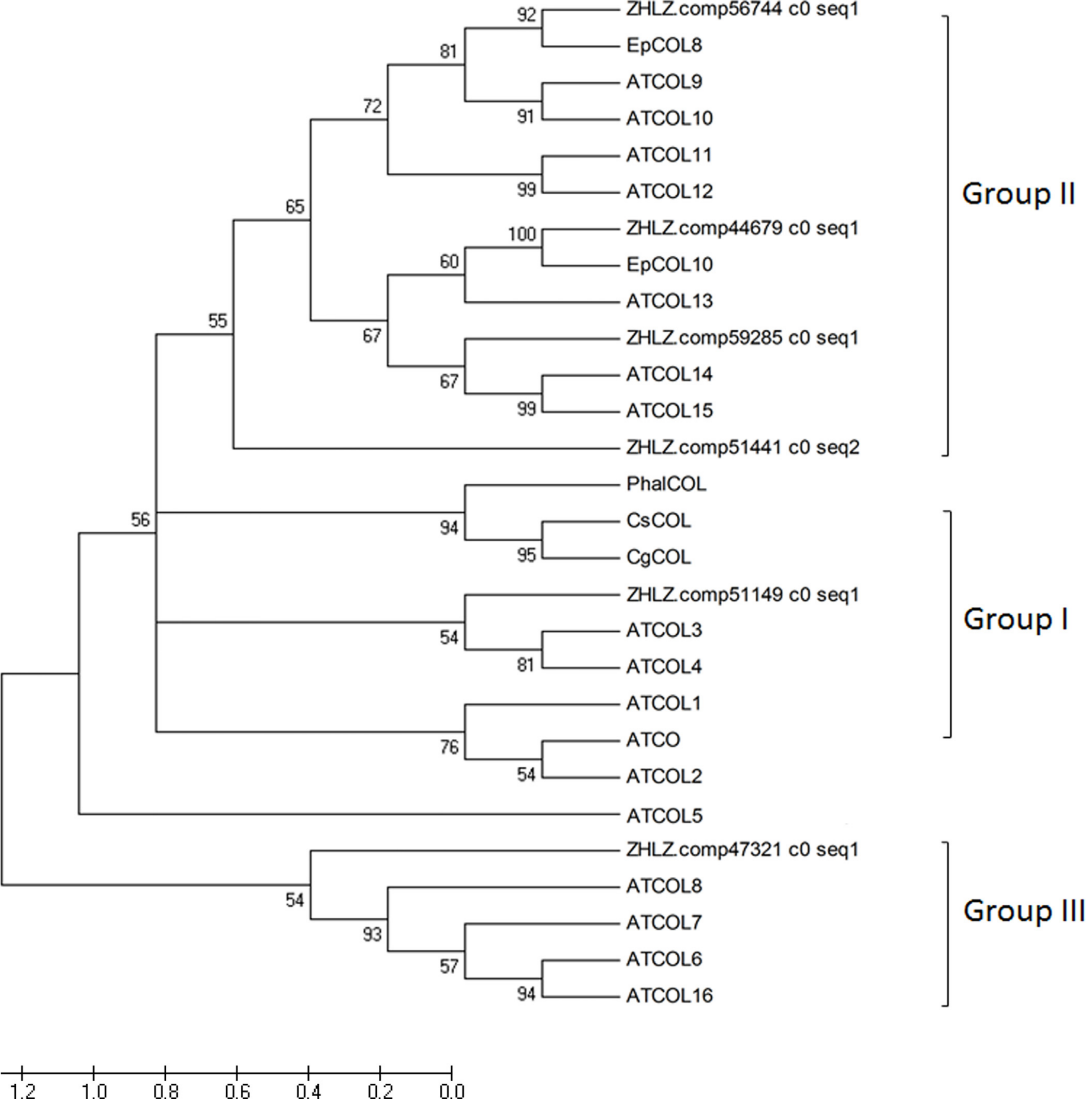
Phylogenetic analysis of the CONSTANS-like proteins from different plant species. Amino acid sequences were aligned by the ClustalW 2.0, and phylogenetic relationships were reconstructed using a maximum-likelihood (ML) method in PHYML software with JTT amino acid substitution model. Bootstrap values for 1,000 replicates were used to assess the robustness of the trees. Previously published plant MADS-box protein sequences were retrieved from GenBank database. (AtCO: NP_197088, AtCOL1: NP_197089, AtCOL2: NP_186887, AtCOL3: Q9SK53, AtCOL4: Q940T9.2, AtCOL5: Q9FHH8, AtCOL6: Q8LG76, AtCOL7: Q9C9A9, AtCOL8: Q9M9B3, AtCOL9: NP_001118599, AtCOL10: Q9LUA9, AtCOL11: O23379, AtCOL12: Q9LJ44, AtCOL13: O82256, AtCOL14: O22800, AtCOL15: Q9C7E8, AtCOL16: Q8RWD0, PhalCOL: FJ469986, CsCO: GU168786. EpCOL8: KC836891.1, EpCOL10: KC836893.1)

**Fig 7:**
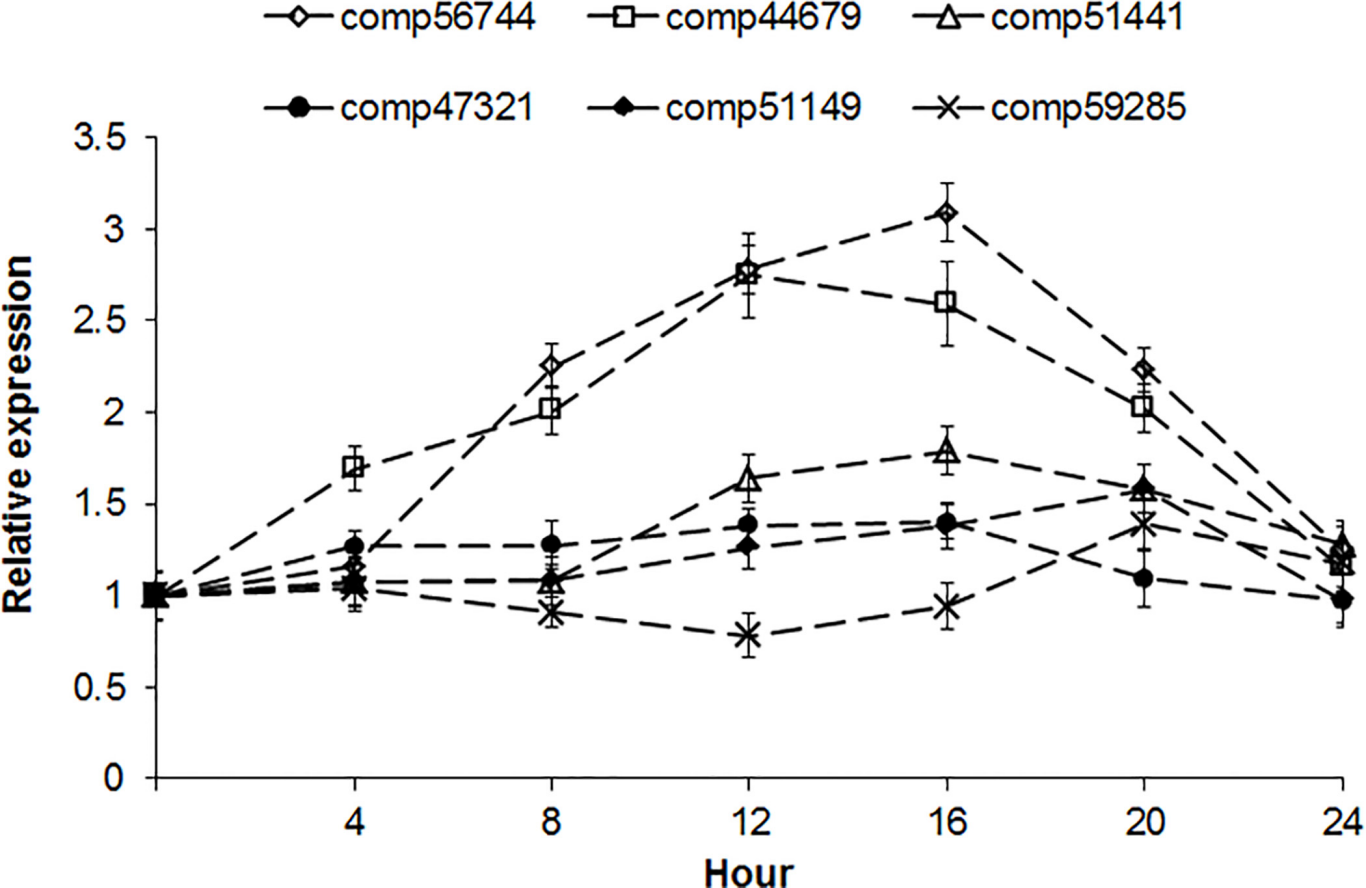
Accumulation of *Cymbidium ensifolium COL* genes in LD conditions. Leaves from one-year-old *C*. *ensifolium* plants were collected in 4-h intervals for 24 h after the start of light exposure in LD conditions (16-h light /8-h dark).

The *COL* genes belonging to different subgroups are expected to perform distinct biological roles, but only a few of them can be regulated by the circadian clock and light, and plays a central regulatory role in the photoperiod pathway [36]. Here, we present a further determination of six *COL* genes as shown in Figs 6 & 7. When compared to other *COL* genes from *Arabidopsis* and other orchid plants including *Phalaenopsis hybrid*, *Cymbidium sinense* and *Erycina pusilla*, these six selected genes can be divided into three subgroups: unigene comp51149 in group I was clustered with CsCOL, and PhalCOL, as well as *Arabidopsis* AtCO/AtCOL1/AtCOL2. Group II included unigenes comp56744, comp44679, comp59285, and comp51441. They are closely related to *Erycina pusilla* EpCOL8/EpCOL10 and *Arabidopsis* AtCOL10/AtCOL13/AtCOL14/AtCOL15. Comp47321 was assigned to group III and clustered well with AtCO6/AtCOL7/AtCOL8. The temporal expression of them also indicated three patterns of light regulation (Fig 7). Genes that belong to the Type I expression pattern, including unigenes comp56744, comp44679, and comp51441, were abundant in the light. Type II genes, which included comp47321 and comp51149 were expressed constitutively in the light or dark. Contrarily, the expression of Type III gene comp59285 was increased in the dark, but repressed in the light. These expression patterns may correlate with the regulatory regions or biological functions of them, and further investigations using these genomic sequences will provide more insight into the photoperiodic control of flowering in the orchid plants.

### DGE sequencing and analysis

To identify the transcriptional responses in individual flower organs, we constructed independent sepal, petal, labellum, and gynostemium DGE libraries, and sequenced them using the Illumina HiSeq 2500 sequencing platform. For each library, the total number of counts for each read was determined, and all the reads were mapped back to the reference transcriptome. The DGE sequencing quality evaluation and alignment statistics are shown in Table 5, which revealed that the clean reads in each sample ranged from 26 to 30 million. Among the clean tags, 28,314,897, 28,885,091, 26,626,616, and 20,985,335 sequences were corresponding to 91.15%, 92.47%, 90.35%, and 79.82%, respectively, of the sepal, petal, labellum, and gynostemium transcriptome libraries, respectively. Thus, good coverage of the transcript profiles was provided.

**Table 5:** Summary of DGE sequencing quality and alignment.

	Sepal	Petal	Labellum	Gynostemium
**Total reads**	31,063,650(100%)	31,237,880(100%)	29,464,502(100%)	26,291,414(100%)
**Total mapped**	28,314,897(91.15%)	28,885,091(92.47%)	26,626,616(90.35%)	20,985,335(79.82%)
**Multiple mapped**	3,882,139(12.50%)	3,057,936(9.79%)	3,595,240(12.20%)	5,328,168(20.27%)
**Uniquely mapped**	24,432,758(78.65%)	25,827,155(82.68%)	23,031,376(78.15%)	15,657,167(59.55%)
**Read1 mapped**	14,166,443(45.60%)	14,454,262(46.27%)	13,321,458(45.20%)	10,499,498(39.94%)
**Read2 mapped**	14,148,454(45.55%)	14,430,829(46.20%)	13,305,158(45.15%)	10,485,837(39.88%)
**Reads map to’+’**	14,170,934(45.62%)	14,437,837(46.22%)	13,337,405(45.26%)	10,524,193(40.03%)
**Reads map to’-**	14,143,963(45.53%)	14,447,254(46.25%)	13,289,211(45.09%)	10,461,142(39.79%)
**Duplication**	12,403,029(39.93%)	15,231,879(48.76%)	11,439,220(38.82%)	2,605,567(9.91%)

### Gene expression profiling among different floral organs

**Fig 8:**
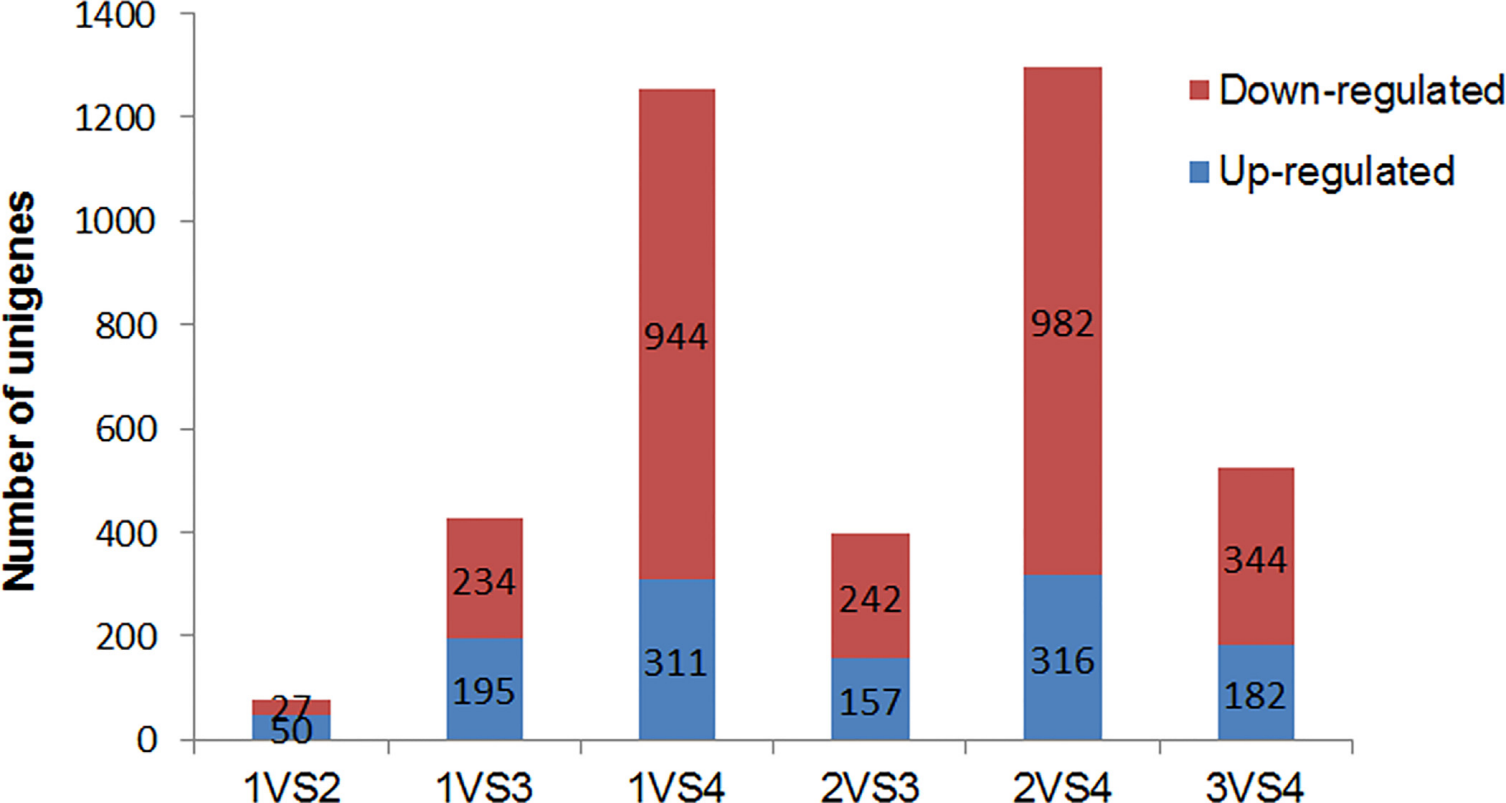
Transcripts differentially expressed between different floral organs. Up- and down-regulated transcripts were quantified. The results of six comparisons between each two samples are shown. 1, sepal; 2, petal; 3, Labellum; 4, gynostemium.

To characterize the gene expression profile of each floral organ, we determined the differentially expressed unigenes between every two samples using the RPKM method with an algorithm developed by Audic et al [39]. From the six pairwise comparisons among sepals, petals, labellum and gynostemium, we found a total of 3,994 unigenes had significant changes in expression, ranged from 77 to 1,298 in different pairwise comparisons. The largest differences occurred between the petals and the gynostemium, and there were 982 and 316 transcripts up- and down-regulated in the gynostemium, respectively (Fig 8). The smallest difference occurred between sepals and petals, in which only 77 differentially expressed tags were identified. The differences among the other comparisons ranged from 399 to 1,255 transcripts.

To determine the putative biological functions of these differentially expressed unigenes, we mapped them to every term in the GO database (http://www.geneontology.org/) and 3,292 unigenes were annotated. This analysis revealed that 41 GO terms showed significantly different expression levels among different floral organs. These GO terms included ‘structural constituents of ribosome’ and ‘ATP binding’ in the ‘molecular function’ category, ‘ribosome biogenesis’, ‘translation’, and the ‘oxidation-reduction process’ in the ‘biological process’ category, and ‘membrane-bounded vesicle integral to membrane’, and ‘ribosome’ in the ‘cellular component’ category (S6 Table). Most of the enrichment occurred in the gynostemium as opposed to the other three libraries, and these genes were mainly correlated to ‘binding’, ‘membrane-bounded organelles’, and ‘organelle parts’, which correlated well with the genes identified in the stamen or pollen transcriptomes of other plants.

For the pathway enrichment analysis, we mapped those differentially expressed unigenes to terms in the KEGG database and searched for KEGG terms that were significantly enriched compared with the transcriptome background. In total, 1,734 unigenes were assigned to 176 KEGG pathways. The pathways that were most represented by the unigenes were ‘ribosome’ (72), followed by ‘oxidative phosphorylation’ (29), ‘Phenylpropanoid biosynthesis’ (9), ‘Gluconeogenesis’ (8), ‘protein processing in endoplasmic reticulum’(8), and ‘plant-pathogen interaction’(7) (S7 Table). Consistent with the result from the GO annotation, most differences occurred between the gynostemium and the petal, and the functional transcripts involved in the ‘ribosome and oxidative phosphorylation’ pathway accounted for a large proportion. Thus, further studies on the precise roles of these genes will provide new insights into the flower patterning mechanism of *C*. *ensifolium*.

### Differentially expressed genes specific for each floral organ

To further identify the genes specifically responding to individual floral organ development, we selected genes that showed at least a fourfold higher expression after filtering statistically significant expression differences using the p-value threshold of 0.05. Moreover, genes that were up-regulated in more than one of the organs were not included. In total, we identified 10 petal-, 5 sepal-, 279 labellum- and 764 gynostemium-specific genes. Most of the enriched transcripts were connected to cell structures, plant metabolic process and the oxidation-reduction process, including cDNAs encoding homologs of cell wall-related genes, such as cellulose synthase-like proteins and cell wall-associated hydrolase family members, membrane associated proteins, such as membrane protein, photosystem II thylakoid membrane protein, and MATE efflux membrane protein, and a number of genes involved in secondary metabolism and oxidation-reduction pathways, such as the acetyltransferase, transketolase, and NADP-dependent oxidoreductase (Table 6). The specifically up-regulated expression of these genes strongly suggests putative functions for them in flower organ development and/or differentiation.

**Table 6:** Tissue-specific genes revealed from DGE expression of various flower organs.

**4-fold up in sepals**	
**Sequence ID**	**Putative function**
**Metabolism**	
Comp49718	NADP-dependent oxidoreductase P2 [*Zea mays*]
**Comp60267**	O-acyltransferase WSD1-like [*Glycine max*]
**Comp59584**	endo-beta-1,4-glucanase [*Pyrus communis*]
**Comp45151**	O-methyltransferase [*Ricinus communis*]
**Comp28605**	Putative phospholipase a2 precursor [*Arabidopsis thaliana*]
**Cell structure**	
**Comp55252**	myosin light chain 2 [*Lonomia obliqua*]
**Comp53878**	Alpha-expansin 8 precursor [*Oryza sativa Japonica Group*]
**4-fold up in petals**	
**Sequence ID**	**Putative function**
**Metabolism**	
**Comp58735**	transketolase, chloroplastic-like [*Oryza sativa Japonica Group*]
**Comp58022**	1-D-desoxyxylulose 5-phosphate synthase (DXS) [*Vitis vinifera]*
**Comp56799**	transketolase [*Camellia sinensis*]
**Comp53861**	transketolase isoform 1 [*Camellia sinensis*]
**Comp52466**	sphinganine C(4)-monooxygenase 2-like isoform 1 [*Solanum lycopersicum*]
**4-fold up in the labellum**	
**Sequence ID**	**Putative function**
**Cell structure**	
**Comp64311**	plasma membrane associated protein [*Populus trichocarpa*]
**Comp57083**	leucine-rich repeat transmembrane protein kinase [*Ricinus communis*]
**Comp8922**	putative apolipoprotein A-I precursor [*Meleagris gallopavo*]
**Comp8510**	40S ribosomal protein S10 [*Vitis vinifera*]
**Comp58037**	Cell wall-associated hydrolase [*Solanum lycopersicum*]
**Metabolic process**	
**Comp56608**	3-hydroxyacyl-CoA dehydrogenase [*Arabidopsis thaliana*]
**Comp58442**	transketolase [*Camellia* sinensis]
**Comp48310**	alcohol dehydrogenase [*Solanum lycopersicum*]
**Signal transduction**	
**Comp56125**	NBS-LRR disease resistance protein precursor [*Meleagris gallopavo*]
**Comp33694**	ADP-ribosylation factor, arf [*Riptortus pedestris*]
**4-fold up in the gynostemium**	
**Sequence ID**	**Putative function**
**Cell Structure**	
**Comp58037**	cell wall-associated hydrolase [*Veillonella sp*. 6_1_27]
**Comp57393**	cell wall-associated hydrolase [*Neisseria meningitidis*]
**Comp55155**	cell wall-associated hydrolase [*Neisseria meningitidis*]
**Comp54344**	cellulose synthase-like protein D4-like [*Arabidopsis thaliana*]
**Comp51052**	expansin-A4-like [*Ricinus communis*]
**Comp44366**	YABBY domain transcription factor family protein [*Populus tomentosa*]
**Comp36904**	Protein HIS-71 [*Caenorhabditis elegans*]
**Comp49964**	UPF0497 membrane protein [*Arabidopsis thaliana*]
**Comp49767**	major protein body membrane protein MP27 like [*Capsella rubella*]
**Comp44488**	ABA-induced plasma membrane protein protein [*Arabidopsis thaliana*]
**Comp106967**	putative MATE efflux membrane protein [*Solanum lycopersicum*]
**Comp17934**	60S ribosomal protein L30-like isoform 1 [*Vitis vinifera*]
**Metabolic process**	
**Comp52552**	polygalacturonase precursor-like [*Ricinus communis*]
**Comp7369**	serine acetyltransferase [*Medicago truncatula*]
**Comp57200**	sesquiterpene synthase [*Oryza sativa Indica]*
**Comp36904**	Histone H3.3 [*Oryza sativa Indica*]
**Comp43882**	trytophan synthase alpha subunit, putative [*Ricinus communis*]
**Comp54620**	cytochrome P450 A [*Capsicum annuum*]
**Comp35538**	metallothionein-like protein [*Sorghum bicolor*]
**Comp59258**	heavy metal ATPase [*Populus trichocarpa*]
**Comp102609**	ATP carrier protein 1-like [*Glycine max*]
**Oxidation−reduction process**	
**Comp53444**	stearoyl-ACP desaturase-like protein [*Glycine max*]
**Comp8922**	apolipoprotein A-I [*Lagopus lagopus*]
**Comp48818**	myo-inositol oxygenase [*Solanum lycopersicum*]
**Comp54494**	L-ascorbate oxidase-like protein [*Aegilops tauschii*]
**Comp47117**	seq polyphenol oxidase [*Ananas comosus*]
**Comp49915**	ACC oxidase [*Cymbidium hybrid cultivar*]
**Comp59496**	alcohol dehydrogenase, putative [*Ricinus communis*]
**Comp27255**	AMP dependent CoA ligase, putative [*Ricinus communis*]
**Comp27226**	cytokinin hydroxylase-like [*Cicer arietinum*]
**Transcription**	
**Comp58618**	MADS-box transcription factor [*Oryza sativa Indica*]
**Comp58442**	MADS-box transcription factor [*Oryza sativa Indica*]
**Comp52003**	MADS-box transcription factor [*Oryza sativa Indica*]
**Comp50822**	MADS-box transcription factor [*Oryza sativa Indica*]
**Comp60086**	R2R3-MYB transcription factor [*Oryza sativa Indica*]
**Comp52709**	probable WRKY transcription factor [*Oryza sativa Indica*]
**Comp1478**	heat shock 70 kDa protein cognate [*Glycine max*]
**Signal transduction**	
**Comp44110**	phytochrome C [*Oryza sativa Indica*]
**Comp54413**	ras-related protein RABA1f-like [*Linum usitatissimum*]
**Comp56125**	NBS-LRR disease resistance protein precursor [*Glycine max*]
**Comp45941**	CBL-interacting protein kinase [*Oryza sativa Indica*]
**Comp43483**	ralf-like 22 protein [*Vitis vinifera*]

**Fig 9:**
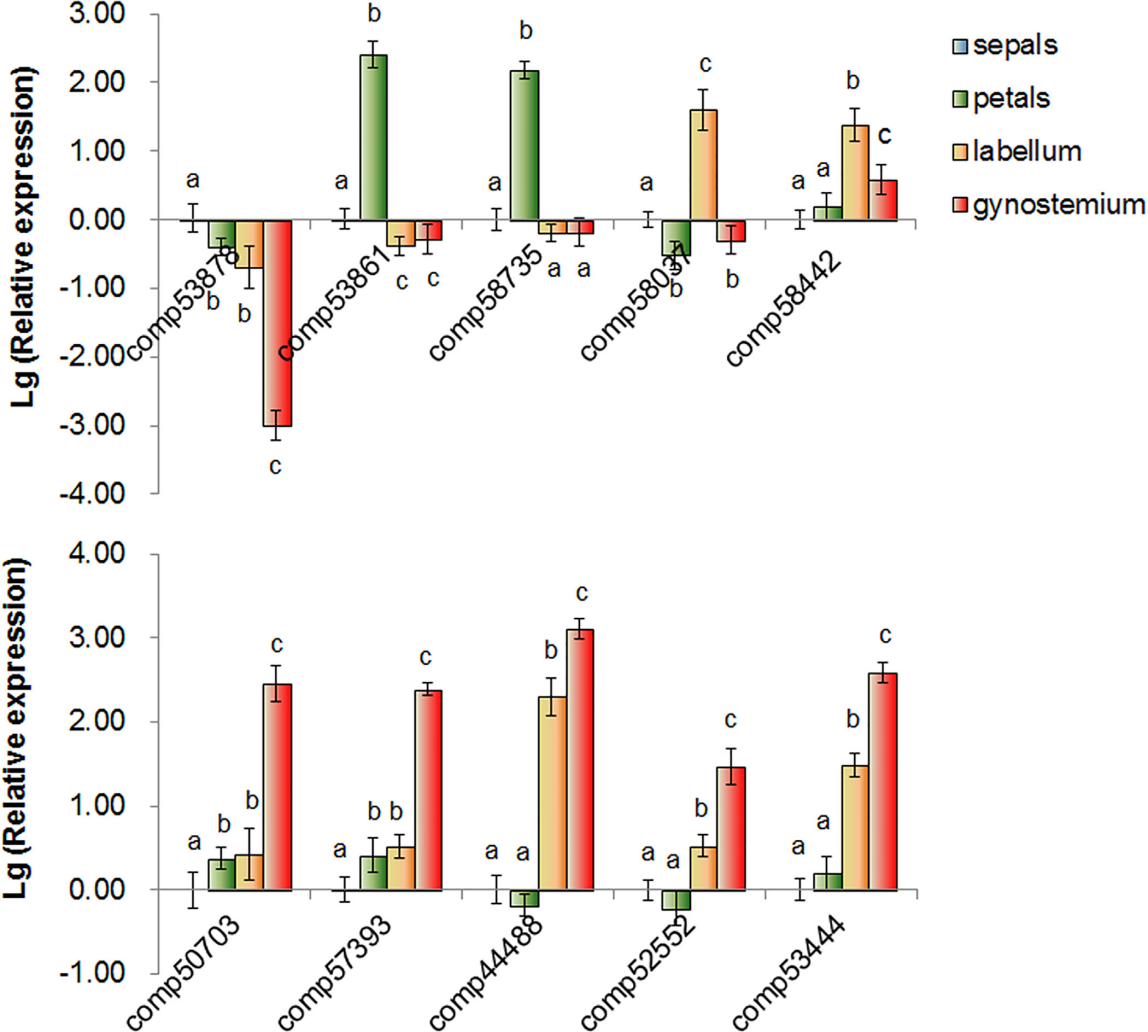
The quantitative RT-PCR analysis of gene expression in sepals, petals, labellums and gynostemiums. The y-axis indicates fold change in expression among the samples. The Lg (Relative Quantitation) of the genes in the sepals was calibrated as zero. Error bars indicate the standard deviation of the mean (SD) (n = 3). Three replicates were analyzed, with similar results. a, b, and c, d, one way ANOVA with Bonferroni multiple comparison test significant at P<0.05 between two of the individual floral organs sepal, petal, Labellum, gynostemium.

To validate the discovery methods, we randomly chose 10 unigenes showing differential expression among these four floral organs and designed primers for quantitative RT-PCR. An EST-encoding ubiquitin was used as an internal control, to which gene expression was normalized. The same mRNA flower samples used in the microarray analysis served as templates. The results exhibited differential expression among the four libraries and were identical to those obtained by DGE expression profiling (Fig 9).

### Differentially expression of transcription factors

**Fig 10:**
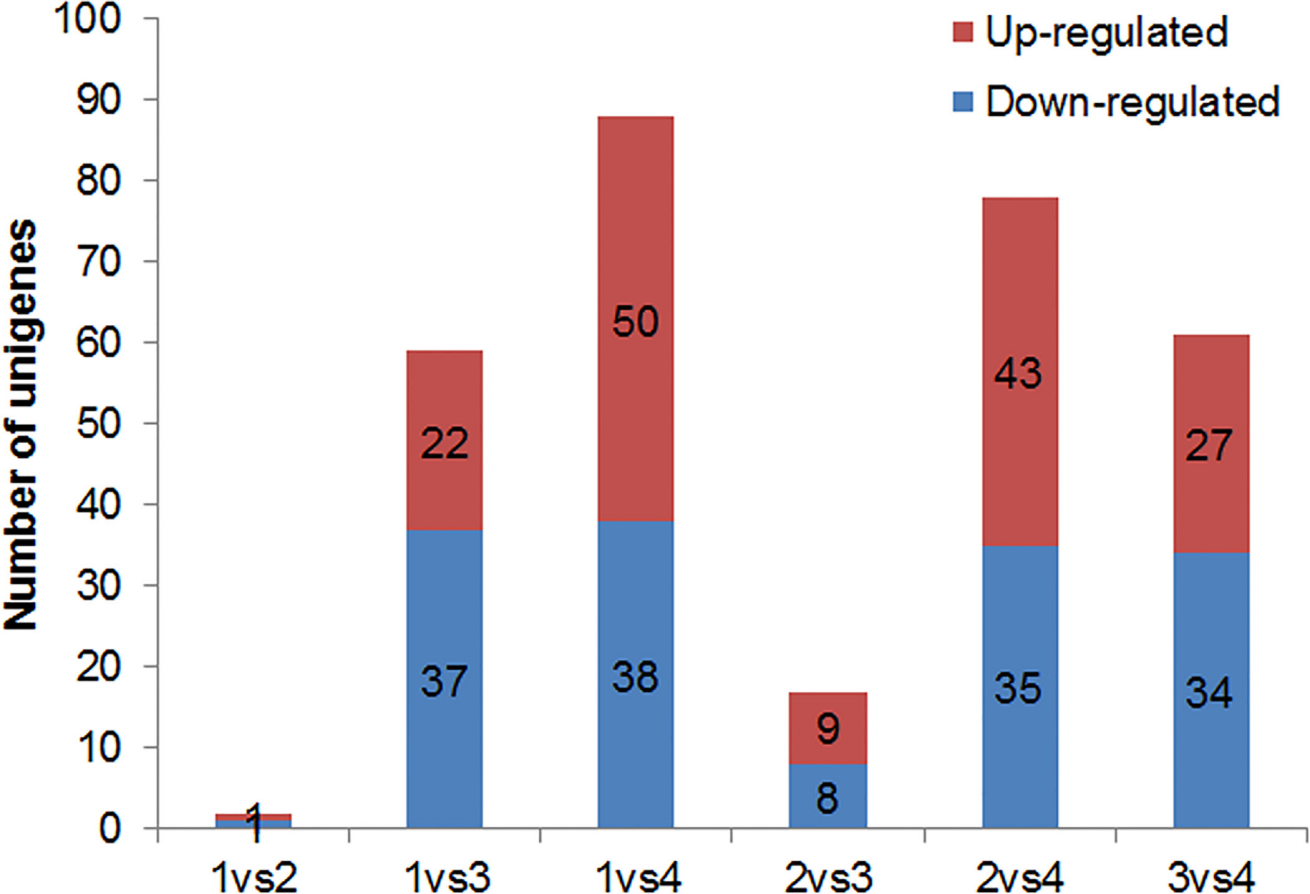
Transcription factors differentially expressed among different floral organs. Up- and down-regulated transcripts were quantified. The results of six comparisons between each two samples are shown. 1, sepal; 2, petal; 3, Labellum; 4, gynostemium.

The transcription factors play a critical role in the control of plant reproductive development. In our study, a total of 4,632 putative transcription factors were detected in the floral transcriptome of C. *ensifolium*. DGE expression analysis revealed 126 of them were differently expressed in different floral organs, suggesting a potential role of them in the regulation of different floral organ development. As shown in Fig 10, there are 2 to 88 transcription factors had significant changes in expression in the pairwise comparisons among sepals, petals, labellum and gynostemium. Typically, the gynostemium contains the highest number of differentially expressed genes, representing the most specialized reproductive organ of the orchid flower.

**Fig 11:**
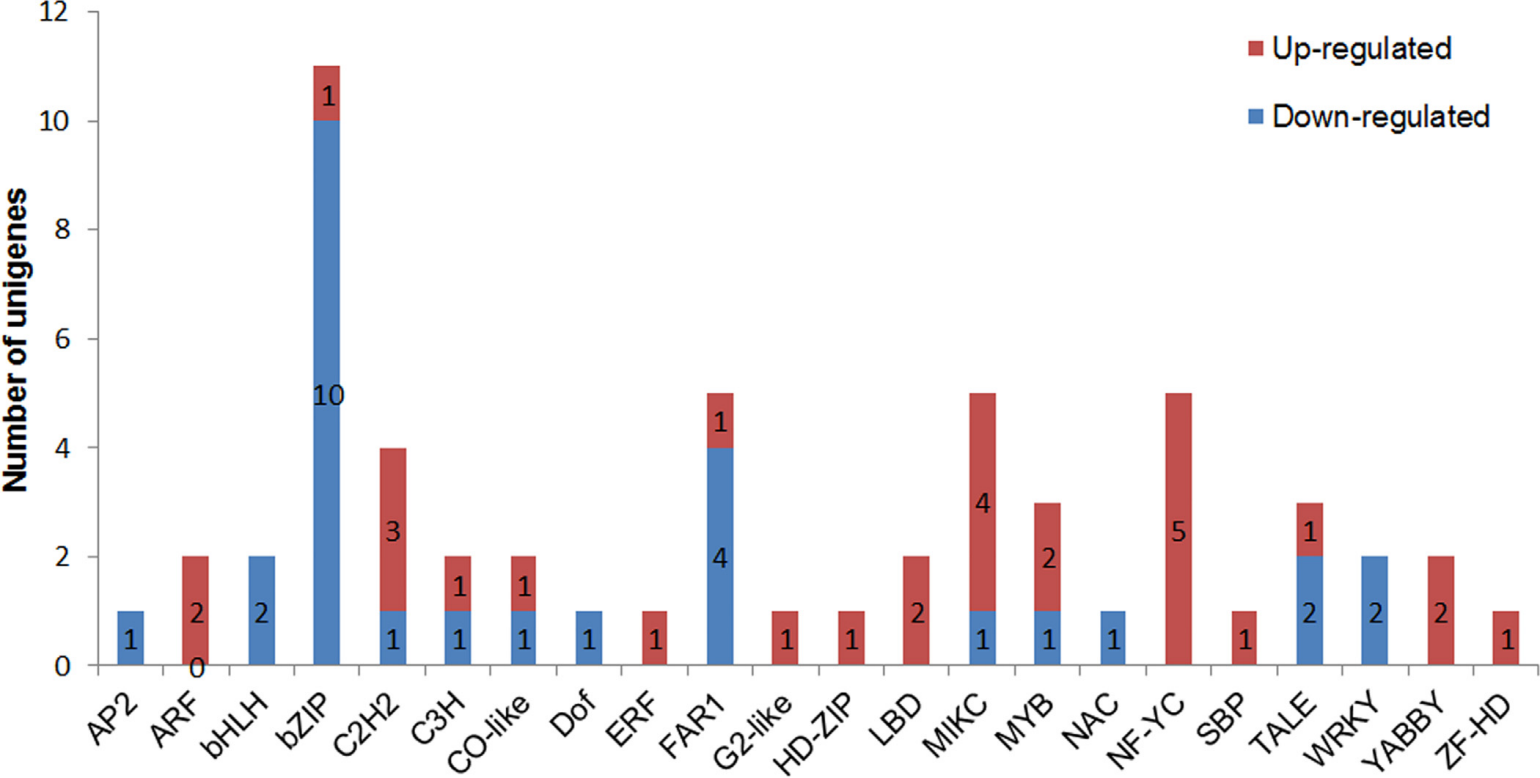
Transcription factors correlated with the development of the gynostemium. Up-(red) and down-regulated (green) in the gynostemium as opposed to other DGEs were quantified and annotated with the plant transcription factor database.

We next concentrated on the transcription factors responding to the gynostemium development and found 30 and 28 transcription factors specifically up- and down- regulated in the gynostemium, respectively, as opposed to the other three libraries. Among these genes, the expression of 5 NF-YC homologs, 4 MIKC-type MADS-box genes, 2 YABBY and 2 MYB transcription factors were increased by more than 4-fold; whereas 10 unigenes with bZIP-like sequences, 4 unigenes with FAR1-like sequences, 2 unigenes with LBD sequences, and 2 unigenes with WRKY-like sequences showed significantly lower abundance in the gynostemium (Fig 11 and S8 Table), suggesting a crucial role of them for floral development. Our work represents the first identification of TFs responding to individual floral organ development, and should be useful for understanding the floral patterning regulation of *C*. *ensifolium*.

### Differentially expression of floral homeotic genes

**Fig 12:**
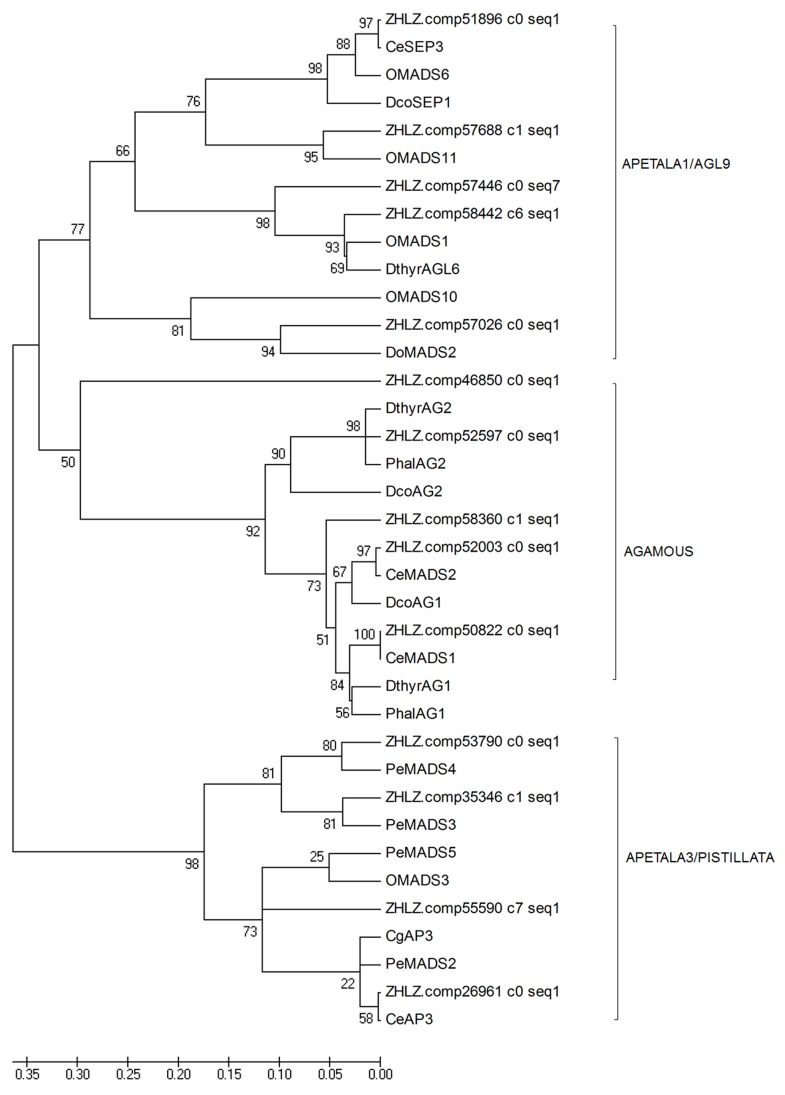
Phylogenetic analysis of the MADS-box genes differentially expressed among different floral organs. Amino acid sequences were aligned by the ClustalW 2.0, and phylogenetic relationships were reconstructed using a maximum-likelihood (ML) method in PHYML software with JTT amino acid substitution model. Previously published plant MADS-box protein sequences were retrieved from GenBank database (PeMADS2: AAR26628, CeAP3-like1: AFH66788, CeAP3-like2: AFH66787, CgAP3:ADI58460, CeMADS2: ADP00516, CeMADS1: ADP00515, CePI-like: AFH66786, OMADS11: ADJ6724, OMADS6: ADJ67238, OMADS1: ADJ67237, OMADS10: ADJ67240, OMADS2: AIJ29175, OMADS3: AAO45824, OMADS4: AIJ29176, DthyrAG1: AAY86364, DthyrAG2: AAY86365, PhalAG1: BAE80120, PhalAG2: BAE80121, PeMADS2: AY378149, PeMADS3: AY378150, PeMADS4: AY378151, PeMADS5: AY378148).

Floral homeotic genes dominantly control the organ identity in developing flowers [18–24]. Most of these genes belong to the MIKC-type MADS-box gene family and can be categorized into several distinct gene groups: APETALA1/AGL9, APETALA3/PISTILLATA, and the AGAMOUS groups [18–24]. From the digital gene expression profiling, we found 16 MADS-box genes differentially expressed among the sepals, petals, labellum and gynostemium (S9 Table). Phylogenetic analysis demonstrated 14 of them clustered well with the floral homeotic genes isolated from other orchid species. Among them, five genes fall well into the APETALA3/PISTILLATA group, four genes belong to the AGAMOUS group and other five were classified to the *APETALA1/AGL9* gene group (Fig 12).

**Fig 13:**
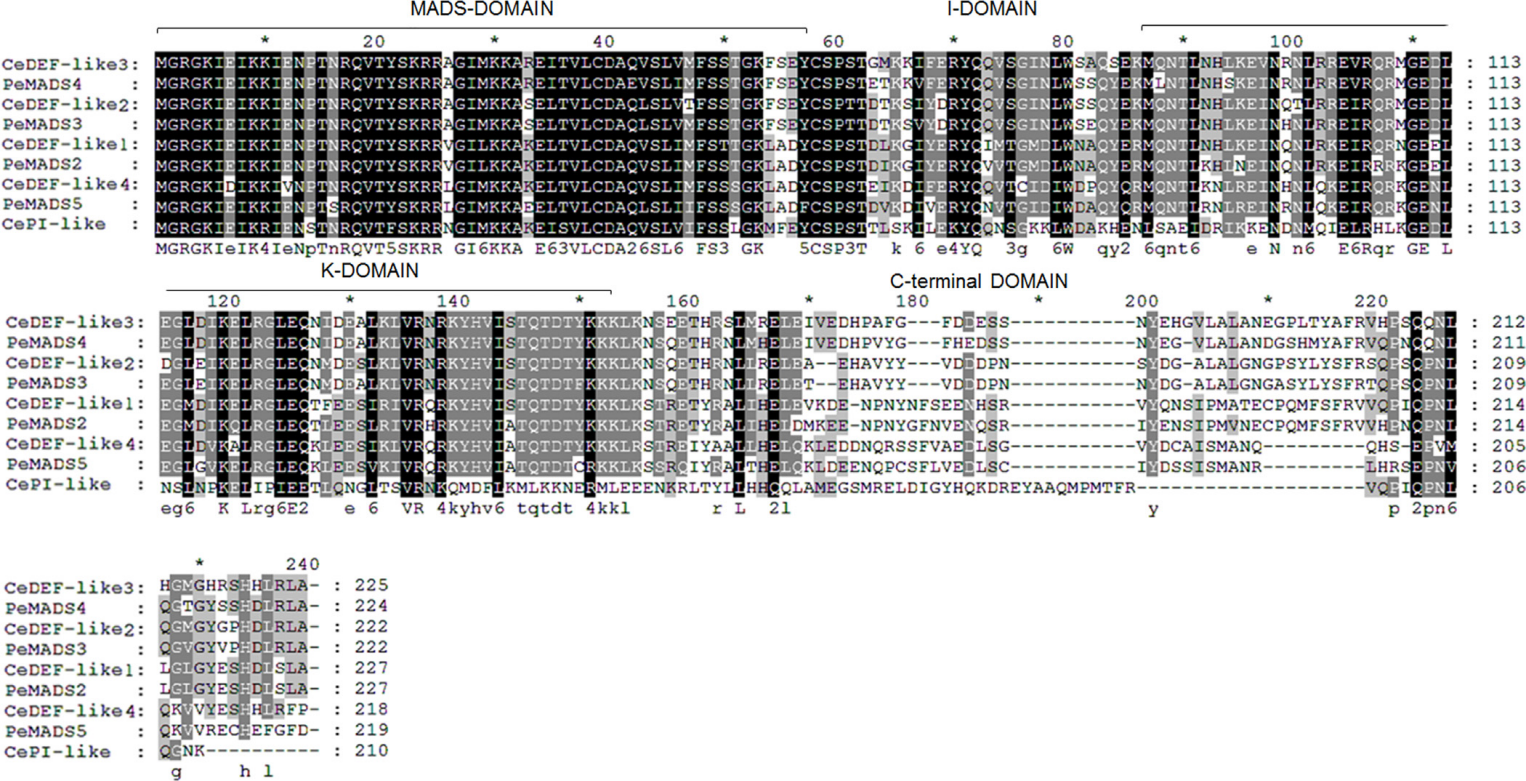
Sequence comparison of the DEF-like genes of *C*. *ensifolium* and *P*. *equestris*. Amino acid sequences were aligned by the ClustalW 2.0. The MADS-, I-, K-, and C-domains are indicated on top of the column.

The topology of the phylogenetic tree indicated that four of the MADS-box paralogs, comp26961, comp35346, comp53790 and comp55590 constituted a well-supported subclade of B-class *DEF*-like gene of *C*. *ensifolium*, as were evident in *Phalaenopsis* [18]. To further characterize these four *DEF*-like paralogs in *C*. *ensifolium*, complete sequences of them were identified by 5’RACE, and named as *CeDEF*-like1 (referring to comp26961), *CeDEF*-like2 (comp35346), *CeDEF*-like3 (comp53790), and *CeDEF*-like4 (comp55590). Conceptual translation of the ORFs yielded proteins of 227 aa, 222 aa, 225 aa, and 218 aa respectively. Multiple sequence alignments with the DEF-like subclade proteins in *Phalaenopsis* demonstrated that all of the four CeDEF-like proteins have a typical MIKC-type domain (Fig 13), and shared 62–79% similarity.

In addition to the four *DEF-like* genes, two *AGOUGOUS*-like C-class genes and two *APETALA*-like E-class genes were also identified in our study. Among them, the full-length cDNAs of C-class gene comp50822 and comp52003 were identical to *CeMADS1* and *CeMADS*2 respectively, consisting well with the previous reported that showed *CeMADS1* and *CeMAD*S2 function in the regulation of reproductive organ development [27]. The E-class gene comp57688 and comp58442 were indicated as the orthologs of *PeSEP1* and *PeSEP3* respectively (Fig 12 and S3 Fig). Amino acid sequences of their proteins shared 67–83% identity and 79–87% similarity with PeSEP1 and PeSEP2, respectively, and possessed the conserved MIK domain and a divergent C-terminal domain with the conserved SEP I and SEP II motifs (S3 Fig).

### Confirmation of differential expression of floral homeotic genes

**Fig 14:**
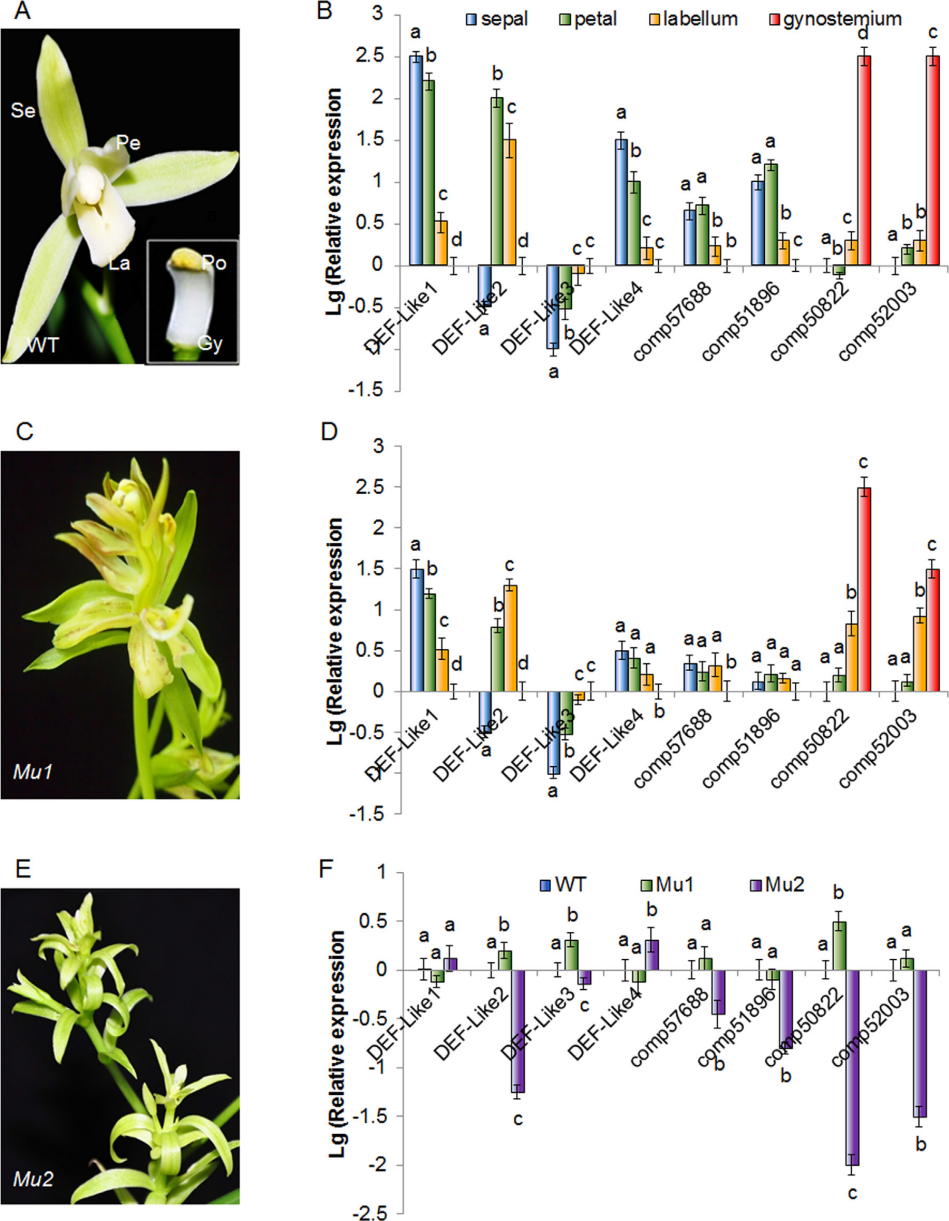
Flower morphology and expression levels of the MADS-box genes in *Cymbidium ensifolium* ‘tianesu’ (WT, A&B), multi-tepal mutant (*Mu1*, C&D) and non-gynostemium mutant (*Mu2*, E&F) Se, sepal primordium; Pe, petal primordium; La, Labellum; Gy, gynostemium; Po, pollinia. The y-axis indicates fold change in expression among the samples. The ubiquitin gene served as the internal control. Error bars indicate the standard deviation of the mean (SD) (n = 3). Three replicates were analyzed, with similar results. a, b, c, and d, one way ANOVA with Bonferroni multiple comparison test significant at P<0.05 between two of the individual floral organs sepal, petal, Labellum, gynostemium.

To validate the results obtained from the digital gene expression and determine the potential roles of the floral homeotic genes referred above, we confirmed their expressions among different floral organs by quantitative RT-PCR. As shown in Fig 14, four paralogs of *DEF-like* MADS-box genes displayed distinctive spatial expression patterns in various floral organs: *DEF-like1* was predominantly expressed in sepals and petals, but showed up weakly in the gynostemium and hardly detected in the labellum, whereas, *DEF-like2* was expressed strongly in the labellum and petals, but to a lesser extent in the gynostemium and not expressed in sepals. By contrast, expression of *DEF-like3* was restricted to the gynostemium and labellum. *DEF-like4* was predominantly expressed in petals, sepals, and hardly detected in the gynostemium. Comp57688 and comp58442, which belong to *AP1/AGL9*-like gene group, were preferentially expressed in sepals, petals and extremely lower in the labellum and gynostemium. On the contrary, the *AGAMOUS*-like genes comp50822 and comp52003 were found to be restricted to the gynostemium (Fig 14A & 14B), consisting well with the previous work that showed C-class gene *CeMADS1/2* were specific to reproductive organ development in *C*. *ensifolium* [27].

Furtherly, we validated the substantially differential expressions of these MADS-box genes in different floral mutant to confirm their functions in floral morphogenesis. Our result indicated that the B-class gene *DEF-like1* was expressed much higher in the labellum of the multi-tepal mutant, whereas expression of *DEF-like4* was extremely eliminated in petals and sepals compared with those in the wild type. These results were in agreement with the findings from other orchid species that showed the petaloid character of the outer tepals is due to heterotopic expression of class B genes such as *HrDEF*, *DcOAP3A*, *PeMADS2* and *PeMADS5*, and *OMADS3* in *Habenaria radiata*, *Dendrobium crumenatum*, *Phalaenopsis equestris* and the *hybrid Oncidium* "Gower Ramsey", respectively [18, 19, 24, and 40]. In addition, the preferentially expression of *SEP-like* genes in the tepals were eliminated in the multi-tepal mutant compared to those in the wild type, and the expression of C-class genes comp50822 and comp52003 were also extended to the labellum of multi-tepal mutant (Fig 14C & 14D). By contrast, we found the overall expression level of the genes which preferentially expressed in reproductive organs, including comp53790, comp50822 and comp52003, were extremely lower in the flower of the non-gynostemium mutant that exhibited petal-to-sepal transformations (Fig 14E & 14F), likely due to the absence of normal petals and gynostemium in the center of the flower. The distinctive expression patterns of these MADS-box genes indicated their divergent functions in controlling floral organ development in different whorls, which have evolved into petaliod perianth (sepals and petals) and reproductive-related organs (labellum and gynostemium). Thus, the data generated in this study is sufficient to be used as a tool to investigate the role of some specific floral homeotic genes that show comparative expression levels among different floral organs.

## Conclusion

High-throughput sequencing and *de novo* assembly were performed for *C*. *ensifolium*. As a result, more than 10 Gb data were obtained and assembled into 111,892 unigenes with an average length of 932.03 base pairs, which represent orthologs of known plant genes, as well as potential new genes. This systems bioinformatics survey, combined with molecular biology analyses, provide a more comprehensive gene information of *C*. *ensifolium* than was previously available, including more functional genes and transcription factors as well as detailed information on the relevant genes involved in flowering and floral development. In this work, we also investigated the gene expression profiling of the individual floral organs through four independent digital gene expression libraries prepared from the sepal, petal, labellum and gynostemium, which revealed 3,994 differently expressed gene tags. Further functional annotation of these tags provided a comprehensive understanding of the transcriptome complexity of floral structures and organ identities. To the best of our knowledge, this study provides a more comprehensive genomic resource and is the first informative EST dataset for individual floral organs of *Cymbidium*. This information broadens our understanding of the mechanisms of floral patterning and contributes to molecular and genetic research in the orchid plants.

## Supporting Information

S1 Fig Metabolic pathway of the circadian rhythm for unigenes by KEGG (JPG)

S2 Fig Phylogenetic analysis of the *COL* genes with its homologues (TIF)

S3 Fig Sequence alignment of SEP- like MADS box proteins with its homologues (TIF)

S1 Table The primers used for Realtime RT-PCR. (TXT)

S2 Table The unigenes involved in the circadian rhythm annotated by KEGG pathway. (TXT)

S3 Table Representative floral homeotic gene including the ABCDE classes. (XLS)

S4 Table A total of 129 MADS-box genes in *C*. *ensifolium*. (TXT)

S5 Table The homology genes of *CONSTANS*-like family in *C*. *ensifolium*. (TXT)

S6 Table Gene set enrichment analysis by GO annotation. (XLS)

S7 Table Gene set enrichment analysis by KEGG metabolic pathway classification. (XLS)

S8 Table Unigenes encoding putative transcription factors associated with the gynostemium development. (XLS)

S9 Table Sequences of the MADS-box genes differentially expressed among different floral organs. (TXT)
