# Supplementary figures and images for: Digital Gene Expression Analysis Based on De Novo Transcriptome Assembly Reveals New Genes Associated with Floral Organ Differentiation of the Orchid Plant Cymbidium ensifolium
Source: PLoS One. 2015 Nov 18;10(11):e0142434. doi: 10.1371/journal.pone.0142434 (PMC4651537; doi:10.1371/journal.pone.0142434)

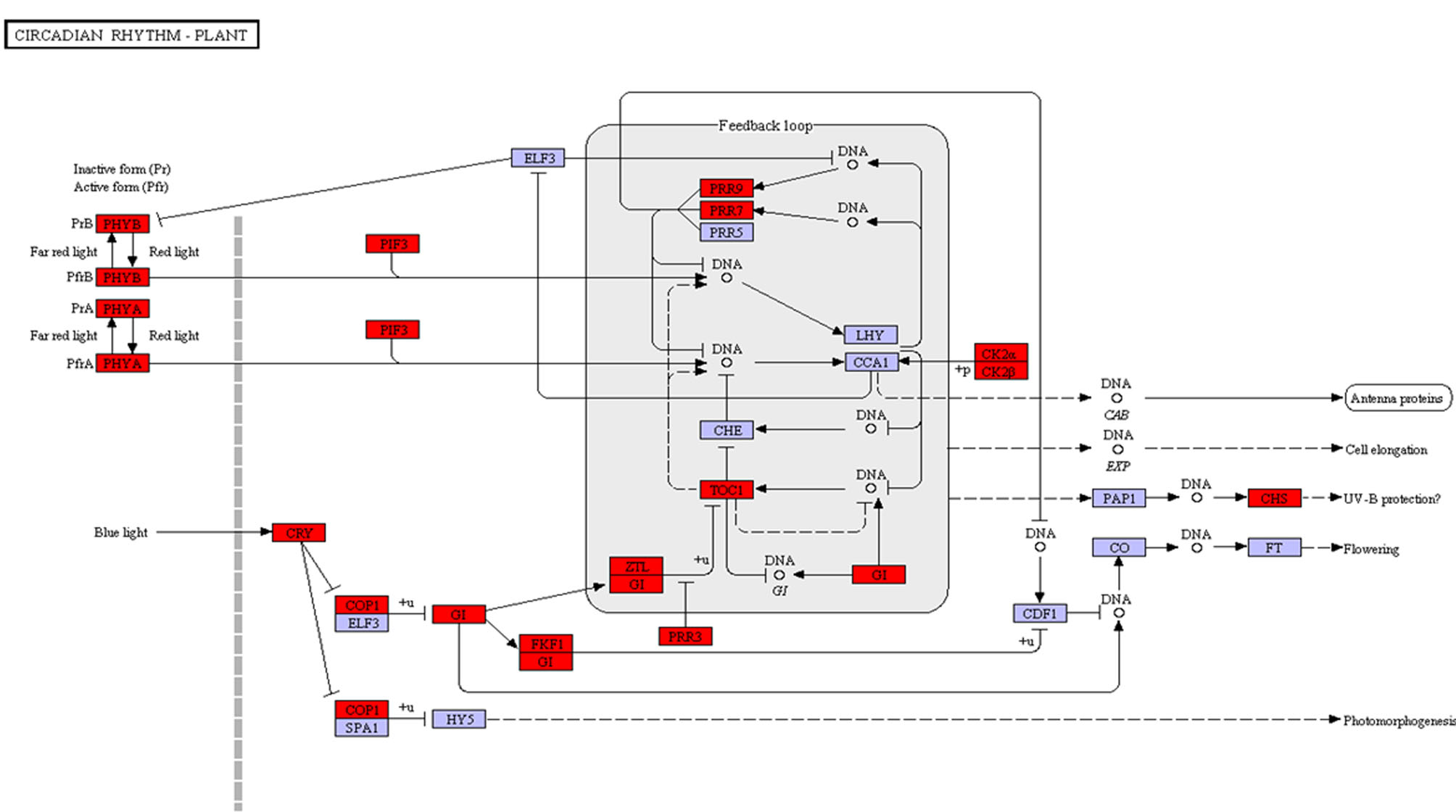

Supplement: S1 Fig — (JPG) [file pone.0142434.s001.jpg]

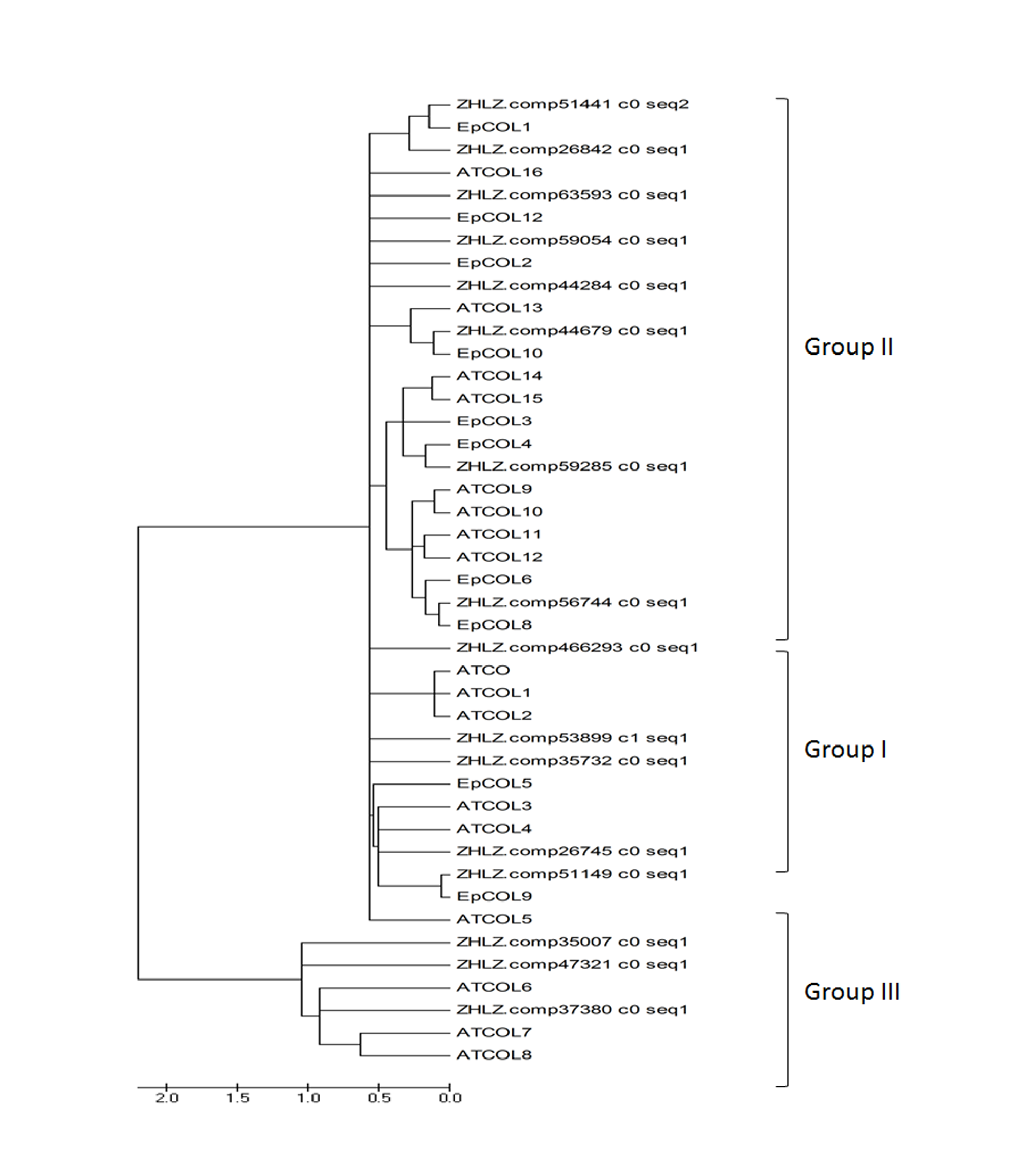

Supplement: S2 Fig — (TIF) [file pone.0142434.s002.tif]

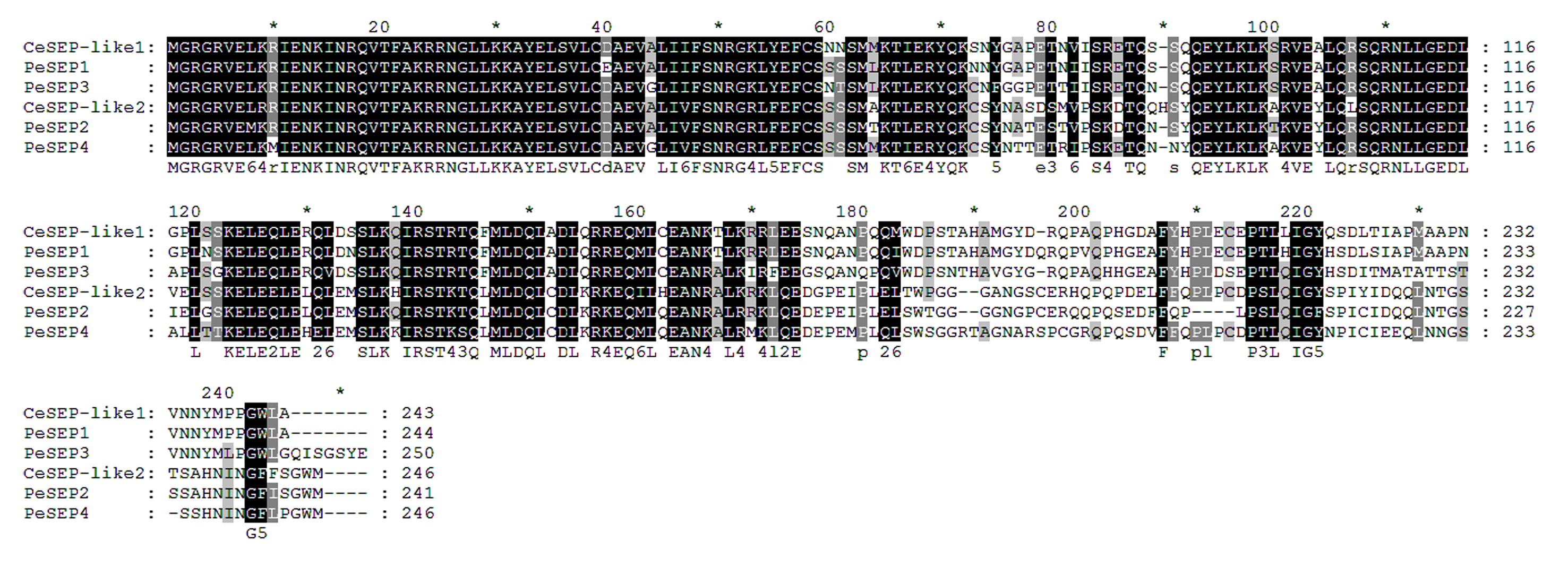

Supplement: S3 Fig — (TIF) [file pone.0142434.s003.tif]
